# Pex1 loss-of-function in zebrafish is viable and recapitulates hallmarks of Zellweger spectrum disorders

**DOI:** 10.3389/fnmol.2025.1634536

**Published:** 2025-11-05

**Authors:** Ursula Heins-Marroquin, Zlatan Hodzic, Beatriz Soares Carneiro da Silva, Agnes Hendriks, Floriane Gavotto, Marc O. Warmoes, Lisa Schlicker, Samy Omri, Christian Jäger, Enrico Glaab, Nancy E. Braverman, Maria Lorena Cordero-Maldonado, Carole L. Linster

**Affiliations:** ^1^Luxembourg Centre for Systems Biomedicine, University of Luxembourg, Esch-sur-Alzette, Luxembourg; ^2^Child Health and Human Development Axis, Research Institute of the McGill University Health Centre, Montréal, QC, Canada; ^3^Division of Medical Genetics, Department of Pediatrics and Human Genetics, McGill University, Montreal, QC, Canada

**Keywords:** zebrafish, Pex1, peroxisome biogenesis disorders, very long-chain fatty acids, phytanic acid, pristanic acid, DHA, disease modeling

## Abstract

Zellweger spectrum disorders (ZSDs) are rare autosomal recessive conditions belonging to the larger group of peroxisome biogenesis disorders. The most prevalent form of ZSD is caused by mutations in the *PEX1* gene, which encodes an AAA ATPase protein. Cells lacking functional PEX1 fail to import proteins crucial for the formation of competent peroxisomes, resulting in aberrant structures called *ghost peroxisomes*. Peroxisome dysfunction leads to the accumulation of compounds that are normally metabolized in this compartment, including very long-chain fatty acids (VLCFAs), pristanic and phytanic acids, as well as deficiency in compounds that are normally formed in this organelle, including docosahexaenoic acid (DHA) and plasmalogen precursors. Patients with a complete lack of PEX1 function develop severe symptoms and have a poor prognosis, with death in the first year of life. In the absence of effective treatments for ZSD, advancing our understanding of this complex multisystem disorder remains essential for uncovering new therapeutic opportunities. To this end, we generated and characterized a zebrafish model with Pex1 loss-of-function. Surprisingly, despite the early onset of disease-relevant features, about 10% of *pex1*^–/–^ zebrafish reached adulthood. However, this resilience was short-lived, as none of the mutant fish survived beyond one year. Histopathological analysis of the liver in adult *pex1*^–/–^ mutants revealed a profound peroxisomal import deficiency and severe vacuolation. Moreover, key metabolic hallmarks of ZSDs, including accumulation of VLCFAs and methyl-branched fatty acids phytanic and pristanic acid, were consistently detected in larval and adult *pex1*^–/–^ mutants. Transcriptomics analysis in *pex1*^–/–^ larvae revealed upregulation of ER-stress responses and pexophagy, as well as dysregulation of neurophysiological processes and visual perception. The latter findings were corroborated by abnormal locomotor behavior in the larvae and by disrupted outer nuclear and retinal layer architecture in adult mutant animals. The described zebrafish *pex1* model provides a versatile *in vivo* platform to uncover novel disease-relevant pathways in ZSD and to investigate the physiological impact of VLCFAs and methyl-branched fatty acids. Its relative tolerance to Pex1 loss-of-function circumvents the early lethality observed in mouse models, enabling the study of ZSD pathophysiology beyond early developmental stages and offering a valuable tool for preclinical therapeutic exploration.

## Introduction

Zellweger spectrum disorders (ZSDs) are rare autosomal recessive conditions belonging to a larger group of disorders known as peroxisome biogenesis disorders (PBDs). The latter are caused by mutations in peroxin (*PEX*) genes, which encode proteins required for the targeting and insertion of matrix and membrane proteins essential for peroxisome assembly and division (Braverman et al., 2013; Hiebler et al., 2014). Peroxisomes are single-membraned organelles present in almost all eukaryotic cells, which host critical metabolic processes such as β-oxidation of very long-chain fatty acids (VLCFAs), α-oxidation of methyl-branched fatty acids, and synthesis of mature bile acids and plasmalogen precursors (Roscher et al., 1985). Consequently, impaired peroxisomal function leads to the accumulation of peroxisomal substrates such as VLCFAs, phytanic and pristanic acid, C27-bile acids, and a reduction in peroxisomal products such as docosahexaenoic acid (DHA, C22:6n-3), C24-bile acids, and plasmalogens (Roscher et al., 1985). These cellular alterations eventually manifest in a wide range of complex clinical symptoms affecting several organs, including the nervous system, the liver, the eyes, and the kidneys. The severity of the disease correlates with the degree of peroxisomal dysfunction, spanning a spectrum from neonatal fatal forms to milder clinical forms with adult onset (Hiebler et al., 2014).

The most prevalent form of ZSD (60–70% of the patients) is caused by mutations in the *PEX1* gene, which encodes a cytosolic AAA ATPase protein (Ebberink et al., 2011; Hiebler et al., 2014). PEX1 assembles into a heterohexameric complex with its partner AAA ATPase PEX6, and the complex is anchored to the peroxisomal membrane via PEX26 (Gardner et al., 2015). The PEX1–PEX6–PEX26 complex mediates the recycling of PEX5 and PEX7, the cytosolic receptors for peroxisomal matrix proteins carrying the peroxisomal targeting signals PTS1 and PTS2, respectively (Soliman et al., 2018). Cells lacking functional PEX1 are unable to import matrix enzymes into peroxisomes due to impaired recycling of ubiquitinated PEX5, leading to a failure in forming functional peroxisomes. This leads to increased pexophagy and to the formation of aberrant structures called *ghost peroxisomes*, considered the cellular hallmark of ZSD (Gardner et al., 2018; Soliman et al., 2018). Severe ZSD patients are born with congenital malformations, specifically polymicrogyria, cortical renal cysts, and limited chondrodysplasia punctata. They also present facial dysmorphia, severe hypotonia, and develop hepatic dysfunction, retinopathy, and deafness (Crane et al., 2005). Unfortunately, these patients have a very poor prognosis, with most succumbing within the first year of life due to respiratory infections or intractable epilepsy (Klouwer et al., 2015). The PEX1 G843D variant is the most prevalent disease-associated allele, accounting for approximately 60% of mutant alleles (Ebberink et al., 2011). The latter encodes an unstable, misfolded protein with residual function associated with milder clinical symptomatology that includes sensory loss, neurological phenotypes (ataxia, polyneuropathy, and leukodystrophy), liver dysfunction, adrenal insufficiency, and renal oxalate stones (Waterham et al., 2016). Currently, there are no effective treatments or cures for ZSD, highlighting the ongoing need to better understand its pathophysiology and to explore potential therapeutic strategies.

Mouse models for Zellweger syndrome, the most severe form of ZSD, have been developed by inactivation of *Pex5*, *Pex2*, and *Pex11*β (Baes et al., 1997; Faust et al., 2001; Li et al., 2002). However, similarly to humans, mice lacking peroxisomal function exhibited severe hypotonia at birth and died shortly after birth, challenging post-natal studies. Another murine model for the milder form of ZSD caused by the common human PEX1 G843D variant, also recapitulated the clinical hallmarks of this form of the human disease, including growth retardation, fatty liver, retinopathy, hair cell degeneration, and hearing loss (Hiebler et al., 2014; Demaret et al., 2020; Mauriac et al., 2022).

Here, we successfully generated a Pex1 loss-of-function zebrafish model using CRISPR/Cas9-mediated genome editing. Our novel zebrafish mutant line carries a 17 bp deletion in exon 5 of the *pex1* gene, resulting in a frameshift and early stop codon. Analogous to other Pex loss-of-function zebrafish models (namely *pex2*, *pex5*, and *pex13*) (Takashima et al., 2021; Bhandari et al., 2023; Demers et al., 2023), *pex1*^–/–^ larvae exhibited normal early development until approximately 11–13 days post-fertilization (dpf). At this stage, the larvae started developing liver steatosis with accumulation of polyunsaturated VLCFAs (or VLC-PUFAs), methyl-branched fatty acids (FAs), and triacylglycerols (TGs). Additionally, network enrichment analysis of transcriptomics data from 11 dpf *pex1*^–/–^ larvae indicated the activation of diverse endoplasmic reticulum (ER) stress responses and alterations in neurophysiological processes and visual perception, bile acid metabolism, connective tissue degradation, and sodium transport. Remarkably, despite showing disease-relevant hallmarks early in larval development, 10% of *pex1*^–/–^ mutants reached adulthood at 3 months of age without any overt phenotype. However, this resilience was temporary, as the mutants’ lifespan was drastically reduced, with no *pex1*^–/–^ mutant surviving beyond one year of age. Adult *pex1*^–/–^ zebrafish showed fatty liver with no morphologically detectable functional peroxisomes. Moreover, biochemical analysis of the liver and brain confirmed accumulation of the expected ZSD biomarkers. Finally, histopathological analysis of the adult retina showed a narrowing of the outer nuclear layer (ONL) as well as a clear disorganization of the cones in the retinal layer, which has also been reported in other ZSD animal models (Argyriou et al., 2019).

Overall, our *pex1* zebrafish model recapitulates key metabolic and pathological features observed in ZSD patients. Intriguingly, however, Pex1 loss-of-function zebrafish do not exhibit the early lethality characteristic of mammalian models, but instead mirror phenotypes associated with milder forms of the disease spectrum. This attenuation may offer a distinct advantage for longitudinal *in vivo* studies aimed at dissecting the sequence of pathogenic events, which remains poorly understood in ZSDs. Furthermore, the small size and high fecundity of zebrafish make them well suited for developing drug screening assays, providing a valuable platform to identify urgently needed therapeutic strategies for these deleterious disorders.

## Material and methods

### Ethics statement

The Zebrafish Core Facility at the Luxembourg Centre for Systems Biomedicine (LCSB) is registered as an authorized breeder, supplier, and user of zebrafish with Grand-Ducal Decree of 20 January 2016 and 26 January 2023. All practices involving zebrafish were performed following European laws, guidelines, and policies for animal experimentation, housing, and care (European Directive 2010/63/EU on the protection of animals used for scientific purposes) and following the principles of the Three Rs. Moreover, we carefully comply with the ARRIVE guidelines. Authorization number LUPA 2020/21 allowed the generation and characterization of the *pex1* mutant line, and authorization number LUPA 2017/04 allowed the performance of fin biopsies for genotyping purposes.

### Zebrafish lines and handling

Wild-type (AB) and *pex1* mutant zebrafish lines were kept in the LCSB Aquatic Platform Core Facility (RRID:SCR_025429) according to standard protocols (Westerfield, 2000; Aleström et al., 2020). Zebrafish embryos were obtained by natural spawning and reared in 0.3X Danieau’s solution [17 mM NaCl, 2 mM KCl, 0.12 mM MgSO_4_, 1.8 mM Ca(NO_3_)_2_, 1.5 mM HEPES pH 7.5, and 1.2 μM methylene blue] in 14 h light–10 h dark condition at 28 °C (± 0.5). Adult fish were maintained in 3.5 liter tanks at a stock density of 4–6 fish/liter with the following parameters: water temperature, 28.5 °C (± 0.5); light:dark cycle, 14 h:10 h; pH 7.5; conductivity, 700–900 μS/cm. Fish were fed three to five times per day, depending on age, with granular food (SDS diets) and live feed (*Artemia salina* and/or L-type rotifers). For live imaging, larvae were first anesthetized with buffered 0.008% tricaine methane sulfonate (MS222, Sigma-Aldrich) and then embedded in 2.5% methylcellulose. Euthanasia was performed by an approved method (i.e., overdose of anesthetic) or whenever required in larvae (for metabolomics) by rapid cooling at temperatures of 2–4 °C (approval numbers LUPA 2017/03 and LUPA 2022/06).

### Pex1 sequence alignment

Multiple sequence alignment was performed with the MUSCLE (RRID:SCR_011812) software from EMBL-EBI using the following peroxisome biogenesis factor 1 protein sequences: *Danio rerio* (NP_001164306.1), *Homo sapiens* (NP_000457.1), *Mus musculus* (NP_001280735.1), and *Drosophila melanogaster* (NP_652016.1). Jalview software (RRID:SCR_006459, v2.11.4.1) was used to prepare the alignments for visualization.

### Generation of CRISPR/Cas9 mutants

Candidate guide RNAs (gRNAs) targeting the zebrafish *pex1* gene were identified and selected using the online tool CHOPCHOP (RRID:SCR_015723)^1^. The generation and purification of gRNAs were performed using the Ambion T7 MEGASCRIPT kit and Qiagen PCR Cleanup kit (#28106) as described in Gagnon et al. (2014). Gene-specific oligonucleotides containing the T7 promoter (5′-TAATACGACTCACTATA-3′), the 20 bp target site (5′-AGATGGCTGACAGACCTAGG-3′), and a 23-bp complementary region (5′-GTTTTAGAGCTAGAAATAGCAAG-3′) were annealed to a constant oligo (AAAAGCACCGACTCGGTGCCACTTTTTCAAGTTGATAACG GACTAGCCTTATTTTAACTTGCTATTTCTAGCTCTAAAAC). Microinjections using wild-type (AB) zebrafish and subsequent generation of the mutant line were performed following the same pipeline as described in Heins-Marroquin et al. (2024). 200 ng of *pex1* gRNA (0.5 μl), 200 ng of *slc45a* sgRNA (0.5 μl), 160 ng of Cas9 Nuclease NLS from *Streptococcus pyogenes* (1 μl, NEB), and 0.2 μl phenol red were mixed in an Eppendorf tube and incubated for 5 min at room temperature. The mixture was placed on ice, and 1 nl was injected into one-cell-stage embryos. For confirmation of the mutation, the CRISPR target site was PCR-amplified from genomic DNA of F1 larvae using NEB Phusion polymerase (NEB, #M0530S) and the following primer pair: zf-5-2-s (5′-CAAAGGAGAAAATTGCTGTTCC-3′) and zf-5-2-as (5′-CTCCGTGTCCTTCTCTTTATGG-3′). The mutant band was cloned into the Promega pGEM-T Easy vector (Promega #A1360) and sequenced in both directions using M13 primers. Sequences were aligned to the *pex1* reference gene using the SnapGene (v6.2.2) software. F1 fish carrying the 17 bp deletion were incrossed to obtain stable F2 generations. Genomic DNA from F2, F3, and F4 generation larvae and adult fish was analyzed in the same way as described for the F1 generation.

### Early larval genotyping

The DNA from 4 and 5 dpf larvae was extracted following the protocol described in Zhang et al. (2020). Single larvae were placed in wells containing 40 μl extraction buffer (EB; 30 mM Tris-HCl pH 8, 25 μg/ml Proteinase K) in a 96-well plate, and shaken at 220 rpm for 40 min at 37 °C. The EB (≈40 μl) was transferred into a PCR 96-well plate, mixed with 15 μl lysis buffer (10 mM Tris-HCl pH 8.0, 50 mM KCl, 0.3% Tween 20, and 1 mM EDTA), and incubated at 98 °C for 5 min. 7 μl of this mixture was used for PCR amplification using the DreamTaqTM Green PCR master mix (K1082, Thermo Fisher) and the primer pair (zf-5-2-s/zf-5-2-as). Amplicons were analyzed by electrophoresis in 3.5% agarose gels and larvae were grouped according to their genotype.

### Viability assay

To monitor survival, a pool of 50–57 larvae at 7 dpf were placed in 1 liter plastic cages (Tecniplast) containing 300 to 500 ml system water and fed daily *ad libitum* with rotifers. After 6 days, artemia was included in the diet. Daily visual assessment was performed, and dead or severely cachectic larvae were recorded and removed from the cage (and euthanized if needed).

### Dissection and collection of organs

Adult zebrafish were euthanized in 0.04% MS222 (buffered to pH∼7.5) at 4, 7, or 10 months of age. The total length of each fish was measured from the snout to the border of the base of the tail (without caudal fin) while the fish was lying on its side. Fish were then photographed, carefully placed on a dissection mat, and organs were collected using dissecting forceps and surgical scissors. Organs were briefly rinsed in chilled 1X DPBS (no calcium, no magnesium, Gibco, #14190250), and either fixed or snap-frozen in 2 ml Precellys tubes in liquid nitrogen. The tubes containing the snap-frozen organs were weighed and stored at −80 °C until further processing. Fixation was done in either 2% or 4% paraformaldehyde (PFA, Sigma-Aldrich, #252549) overnight at 4 °C.

### Proteomics

#### Protein extraction from adult zebrafish organs

Proteomic sample preparation was performed using the iST Sample Preparation Kit for Mammalian Tissue (PreOmics) following the manufacturer’s instructions. Livers from 7 month-old female and male zebrafish were dissected as explained above and snap-frozen, along with 10 ceramic beads (1.4 mm, VWR, Cat. No. 432-0356). Single livers were homogenized in 150 μl LYSE buffer (PreOmics) using a Precellys Evolution Homogenizer equipped with a Cryolys Evolution cooling module (Bertin Instruments) at 6000 rpm for 30 s (1×) at approximately 5 °C. After centrifugation, protein concentration was determined using the Pierce^®^ BCA Protein Assay Kit—Reducing Agent Compatible (Thermo Scientific, P/N 23250). Samples were adjusted to 50 μg of total protein in 100 μl LYSE buffer and transferred to LoBind tubes (Eppendorf, # 0030108442). Subsequent sample processing steps were carried out in accordance with the PreOmics protocol, including digestion, clean-up, and peptide elution. Finally, dried peptides were reconstituted in 50 μl of 0.1% formic acid in water containing 10 fmol/μl of PRTC standard (Pierce™ Peptide Retention Time Calibration Mixture, Thermo Scientific, # 88321) prior to LC-MS analysis. The PRTC standard was added to monitor LC-MS performance, including peptide abundance and retention time consistency, across the sequence run. Three biological replicates per genotype were analyzed and each sample was measured in three technical replicates (three LC-MS injections).

#### LC-MS-based analysis of proteins extracted from adult zebrafish organs

Liquid chromatography was performed on a Vanquish Neo UHPLC system (Thermo Scientific) in trap-and-elute mode with a nano-capillary flow regime. Sample was loaded automatically at a flow rate of 60 μl/min in flow control mode. The trap column was flushed in the backward direction using a target trap wash flow of 200 μl/min. Strong and weak wash factors were set to 50 and 10, respectively, with a target trap wash pressure of 800 bar under combined control mode. The injection workflow included needle washes after sample draw, using 0.1% formic acid in water as the weak wash solvent (3 s at 80 μl/s) and 0.1% formic acid in 80% acetonitrile as the strong wash solvent (5 s at 80 μl/s). The wash cycle time was set to fast. The autosampler was maintained at 7 °C, with vial bottom detection enabled. The injection volume was 1 μl. The draw speed was set to 0.2 μl/s with a 2-s draw delay, and the dispense speed was set to 5.0 μl/s; no air gap was applied. Peptide separation was performed using a 50 cm × 75 μm i.d. EASY-Spray™ PepMap™ Neo UHPLC analytical column (Thermo Fisher Scientific, # ES75500PN) operated at 50 °C. A 5 mm × 300 μm PepMap™ Neo trap cartridge (Thermo Fisher Scientific, # 174500) was used for sample pre-concentration. Gradient elution was performed over a total run time of 66.78 min with a nominal flow rate ranging from 0.3 to 0.5 μl/min. Mobile phases consisted of solvent A (0.1% formic acid in water) and solvent B (0.1% formic acid in 80% acetonitrile). The gradient began at 4% B (0.5 μl/min), increased to 4.5% by 0.7 min, and to 5% at 1 min (0.3 μl/min). It continued to 20% B at 38.0 min, 35% at 56.9 min, and 55% at 57.4 min. At 57.4 min, a column wash step was initiated, ramping solvent B to 99% at 0.5 μl/min within 0.5 min, which was then maintained until the end of the run at 66.78 min. Post-run column equilibration was performed using a trapezoidal tandem wash profile under combined control mode (target flow: 2.0 μl/min; target pressure: 1500 bar), with fast column re-equilibration enabled (equilibration factor: 2.0) at 4% solvent B.

Mass spectrometry analysis was performed on an Exploris 240 system (Thermo Fisher Scientific) using Thermo Scientific Xcalibur software (RRID:SCR_014593, v4.7.69.37) and a data-dependent acquisition method optimized for peptide detection. The total method duration was 66.78 min, with peptide acquisition conducted in positive ion mode under a nanoelectrospray ionization (NSI) source. The spray voltage was set to 1,900 V, and the ion transfer tube temperature was maintained at 280 °C. Lock mass correction was applied using EASY-IC™ in RunStart mode. Full MS scans (MS1) were acquired from m/z 375–1,500 at an Orbitrap resolution of 120,000, with an RF lens setting of 70%. The automatic gain control (AGC) target was customized to a normalized value of 300% (absolute AGC: 3.0 × 10^6^), and the maximum injection time was set to automatic. Microscans were set to 1, and data was acquired in profile mode. Advanced peak determination was enabled, and the default precursor charge state was set to 2. Monoisotopic peak selection (MIPS) was used with relaxed filtering when few precursors were identified. Only precursors with charge states from 2 to 5 were selected; undetermined charge states were excluded. A dynamic exclusion window of 45 s was applied after one detection, with a ± 10 ppm mass tolerance. Isotopic peaks were excluded, and only a single charge state per precursor was selected for fragmentation. Data-dependent MS^2^ scans (ddMS^2^) were triggered in a cycle time mode with 1-s intervals between master scans. Fragmentation was performed using higher-energy collisional dissociation (HCD) with a normalized collision energy of 30%. Ions were isolated using a 1.8 m/z window without offset. MS^2^ spectra were acquired in the Orbitrap at a resolution of 15,000, starting at m/z 120. AGC target values for MS^2^ were also customized to 50% (absolute AGC: 5.0 × 10^4^), with automatic injection time and one microscan per event. MS^2^ data were collected in centroid mode.

#### Proteomics data analysis

Label-free quantification (LFQ) was performed in Proteome Discoverer (RRID:SCR_014477, v3.2.0.450, Thermo Scientific) using standard LFQ processing and consensus workflows. MS data were searched against the *Danio rerio* UniProt protein database (TaxID 7955, 2025-02-05, 3614 sequences), supplemented with a custom FASTA containing full-length peroxisome biogenesis (Pex) proteins, many of which are not included in the canonical proteome. Spectral identification was performed with Sequest HT (trypsin/P specificity, up to two missed cleavages). Search settings included static carbamidomethylation of cysteine (+57.021 Da), dynamic oxidation of methionine (+15.995 Da), and N-terminal acetylation (+42.011 Da), with precursor and fragment mass tolerances of 10 ppm and 0.02 Da, respectively. Additional searches allowed deamidation (NQ, +0.984 Da), methionine loss, and N-terminal glutamine to pyro-glutamate conversion. Minimum peptide length was 7 amino acids for primary searches and 6 for extended searches. Peptide-spectrum matches (PSMs) were validated using Inferys Rescoring and Percolator, applying a 1% FDR at peptide and protein levels. Only PSMs of “High” confidence were retained. Protein inference followed the parsimony principle, grouping proteins based on shared peptides, and only master proteins (with the highest number of unique peptides and sequence coverage) were selected for downstream analysis. Proteins required at least one unique peptide for identification. Quantification was performed at the MS1 level using Precursor Ions Quantifier, with normalization to total peptide abundance and scaling mode set to “On All Average.” Protein abundances were calculated by summing intensities for unique and razor peptides; shared peptides were assigned to the protein group with the most unique peptides. Retention time alignment and feature mapping ensured consistency across runs. Abundance ratios between experimental groups were reported as specified in the Proteome Discoverer “Study Definition” tab, where samples are assigned to replicates and conditions. No imputation of missing values was performed. Contaminants were excluded by filtering out entries present in the PD Common Contaminants Library. Only master proteins meeting all criteria were included in differential expression analyses.

### Lipidomics

#### Phospholipid analysis

A pool of 15 larvae at 13 dpf was transferred into 2 ml Precellys tubes (Bertin Corp.), snap-frozen, and stored at −80 °C until further processing. Phospholipid extraction, measurement, and analysis were performed by Lipometrix (Leuven, Belgium) following the protocol described in Heins-Marroquin et al. (2024). Statistical analysis was performed with the GraphPad Prism analysis software (RRID:SCR_002798, v10.2.1).

#### VLCFA extraction from zebrafish larvae and adult organs

Pools of five larvae at 11 dpf or single dissected organs (liver, brain) were transferred to 2 ml Precellys tubes (Bertin Corp.), snap-frozen, and stored at −80 °C until further processing. On the day of extraction, 600 mg of 1.4 mm ceramic beads (Qiagen) were added. Larvae and organs were processed differently.

For larvae: 50 μl MilliQ water and 800 μl acetonitrile (ACN), spiked with internal standards (IS) at a final concentration of 2 μg/ml [IS: tridecanoic-d24 acid (D-4002, C/D/N Isotopes), methyl heptadecanoate-d33 (00889-10MG, Merck) and docosanoic acid-d43 (D-4005, C/D/N Isotopes)], were added while samples were kept on ice. Samples were homogenized using a Precellys24 homogenizer (Bertin Technologies) with two 30 s cycles at 6,000 rpm (0 to 5 °C), with a 30 s pause between cycles. Homogenates (750 μl) were transferred to 1.5 ml Eppendorf tubes and centrifuged at 21,000×*g* for 10 min at 4 °C. Then, 350 μl of the supernatant was transferred to a new 1.5 ml Eppendorf tube, and 45 μl of 5 M HCl was added. The mixture was vortexed at 500 rpm using an Eppendorf ThermoMixer C and heated at 100 °C for 1 h. Tube caps were sealed with cap locks (Biozym) during heating. Next, 380 μl of the hydrolyzed homogenate was transferred to a new 1.5 ml Eppendorf tube prefilled with 340 μl methyl tert-butyl ether (MTBE) and 320 μl MilliQ water, and vortexed for 5 min at 2,400 rpm at room temperature. Phase separation was achieved by centrifugation at 21,000×*g* for 5 min at 4 °C. Finally, 350 μl of the upper (organic) phase was evaporated overnight at −4 °C using a refrigerated centrifugal vacuum concentrator (CentriVap, Labconco).

For adult organs: ACN-IS volumes were adjusted proportionally to tissue weight using the reference ratio “640 μl per 16.9 mg tissue.” Tissue homogenization was performed at 6,000 rpm for 30 s (0 to 5 °C) using a Precellys24 homogenizer, followed by a brief spin-down. To each homogenate, 80 μl of 5 M HCl per 16.9 mg tissue was added, with volumes individually adjusted based on tissue weight. Samples were incubated for 1 h at 100 °C in a ThermoMixer (500 rpm). Next, 650 μl of the hydrolyzed homogenate was transferred to a 2 ml Eppendorf tube prefilled with 580 μl of MTBE and 580 μl of Milli-Q water. Samples were vortexed for 5 min at room temperature, then centrifuged for 5 min at maximum speed in a benchtop centrifuge. 800 μl of the upper (organic) phase was transferred to a new tube containing 800 μl of Milli-Q water, vortexed for 1 min, and centrifuged again. Finally, 300 μl of the clarified upper phase was collected and dried in a SpeedVac. The dried VLCFA extracts were stored at −80 °C until LC-MS analysis.

#### LC-MS-based analysis of VLCFAs extracted from zebrafish larvae and adult organs

Dried VLCFA extracts were reconstituted in 50 μl (larvae) or 100 μl (organs) acetonitrile/isopropanol (ACN/IPA, 70/30), followed by brief vortexing and addition of 15 μl MilliQ water. The reconstituted samples were vortexed for 10 min (ThermoMixer C, 1,400 rpm, room temperature), briefly centrifuged, and transferred to LC-MS vials with micro-inserts. Samples were analyzed with an Agilent 1290 LC coupled to an Agilent 6560 Q-TOF MS system equipped with a Dual Agilent Jet Stream ESI source. The analytical column (Waters Acquity UPLC BEH C18, 1 × 100 mm, 1.7 μm) was maintained at 40 °C. The autosampler was kept at 4 °C and the injection volume was 5 μl. The mobile phases consisted of 10 mM ammonium formate in ACN/H2O (60/40, eluent A) and 10 mM ammonium formate in IPA/ACN (90/10, eluent B). The following gradient was applied with a flow rate of 0.12 ml/min: 0 min, 20% B; 10 min, 99% B; 13 min 99% B; 13.5 min 20% B; and 18 min, 20% B.

MS analysis was performed using electrospray ionization in negative mode (-ESI, capillary voltage of 3.5 kV, nozzle voltage of 0 kV). The following Q-TOF source and MS settings were applied: drying gas temperature, 350 °C; drying gas flow, 10 l/min (nitrogen); nebulizer, 25 psi; sheath gas temperature, 400 °C; sheath gas flow, 12 l/min; fragmentor, 390 V; Oct RF Vpp, 600 V. Full scan spectra were acquired for an m/z range of 60–1,700 (2 spectra/sec), with the Agilent Mass Hunter LC-MS/MS Data Acquisition software (version B.09.00). Manual peak integration was performed using Agilent Mass Hunter Profinder (vB.08.00). Fatty acids were identified by exact mass (mass error ± 5 ppm), isotopic pattern, and retention time (± 0.15 min) matching with standard compounds (Supplementary Data 1). Raw peak area values are provided in Supplementary Data 1. Fatty acid peak areas were normalized to the IS (tridecanoic-d24 acid). For each FA, the IS-normalized levels were further divided by the corresponding average WT value. The resulting ratios were log2-transformed to represent relative abundances in mutant versus WT samples. Heatmaps and bar plots were generated using GraphPad Prism (v10.2.1).

#### VLCFA extraction from dry and liquid zebrafish food and LC-MS analysis

Fish dry food (SDS100 and SDS400) and liquid food (artemia and rotifers) were placed in 50 ml Falcon tubes and lyophilized overnight to remove water. A sample of RG-Complete (micro-algal based food for rotifers) was processed in parallel. 15 mg of dry samples was added to 2 ml Precellys tubes (Bertin Corp.) containing 600 mg of ceramic beads (1.4 mm; VWR, #432-0356), 90 μl of MilliQ water, and 360 μl of ACN with internal standards [Tridecanoic acid-d25 (D-4002), Docosanoic Acid-d43 (D-4005) at a final concentration of 2 μg/ml; Methyl heptadecanoate-d33 (00889-10MG), Stearic acid-d5 (DLM-2712-0.1) at a final concentration of 6 μg/ml] and homogenized at 6,000 rpm for 2 cycles of 30 s at 4*^o^*C in a Precellys24 Homogenizer (Bertin Corp.). After continuous shaking at 2,000 rpm for 15 min at 4 °C, 350 μl of homogenate was transferred into a 1.5 ml amber glass vial and 20 μl of 5 M HCl was added, followed by a 60-min incubation at 100 °C. Samples were cooled down to room temperature before addition of 700 μl MTBE. After vigorous mixing, 450 μl of the hydrolyzed homogenate, reflecting 5 mg of sample dry weight, was transferred to a new amber glass vial, and 450 μl of ACN/H_2_O/MTBE (4/1/10, v/v/v) was added followed by mixing. Another 300 μl H_2_O was added to induce phase separation and, after mixing and centrifugation at 3,000×*g* for 5 min, 675 μl of the non-polar, upper phase was transferred into a new 1.5 ml amber glass vial. The non-polar phase was washed by adding 600 μl of MilliQ water, mixing, and centrifugation. 400 μl of the non-polar, upper phase was transferred to a new glass vial and solvents were evaporated in a CentriVap (Labconco) at 4 °C overnight. Prior to LC-MS analysis, dried total fatty acid extracts were reconstituted in 200 μl reconstitution solution {40% mobile phase B: 10 mM ammonium acetate in 88/10/2 IPA/ACN/H_2_O and 60% mobile phase A: 10 mM ammonium acetate in 40/60 ACN/H_2_O, spiked with 100 ng/ml 12-[[(cyclohexylamino)carbonyl]amino]-dodecanoic acid (CUDA) (Sanbio, #10007923)} and filtered using Phenex-PTFE syringe filters (Phenomenex, #AF0-3202-52).

The analysis of targeted compounds was performed using a Vanquish UHPLC coupled to a Q Exactive HF MS system equipped with a heated ESI source (Thermo Fisher). The column (Waters ACQUITY UPLC BEH C18, 150 × 2.1 mm, 1.7 μm particle size) was maintained at 45 °C. The autosampler was kept at 4 °C and the injection volume was 1 μl. The flow rate was set to 0.2 ml/min and the mobile phases consisted of 10 mM ammonium acetate in 60/40 H_2_O/ACN (Eluent A, pH unadjusted) and 10 mM ammonium acetate in 88/10/2 IPA/ACN/H_2_O (Eluent B, pH unadjusted). The run consisted of a linear gradient from 40% to 100% Eluent B over 10 min, followed by an isocratic delivery of 100% Eluent B over 3 min, returning to 40% B within 0.1 min, and end with isocratic delivery of 40% Eluent B for 6.9 min. The total run time was 20 min per sample. MS measurements were performed using electrospray ionization in negative mode (-ESI) with the following ESI source settings: Capillary Temperature −320 °C; Sheath Gas—30 AU; Aux Gas—10 AU; Spray voltage—2,500 V. Data were acquired from m/z 70 to 600 at a resolution of 60,000 and Microscans were set to 2. The AGC target was set to 1e6 and the maximum injection time was set to 100 ms. Acquired data were analyzed with TraceFinder (5.2 SP1, Build 496). Target compounds were identified by exact mass (mass error ± 5 ppm), isotopic pattern and retention time (± 0.15 min) matching with authentic standards. Absolute quantification was based on integrated peak area of the deprotonated target compound, normalization to internal standards, and standard addition [available standards: C10:0, C16:0, C16:1, C18:0, C18:1, C20:0 (branched), C20:3, C22:0, C22:6, C24:0, C26:0, C32:6].

### Locomotor behavioral assay

Locomotor activity of 7, 11, 13, and 15 dpf larvae was recorded using a DanioVision system equipped with a tapping device and a temperature control unit (Noldus Information Technology). The EthoVision XT 11.5 software (RRID:SCR_000441, Noldus Information Technology) was used to analyze the movement of individual larvae. Motion values were expressed as mean swimming velocity (mm/s). Recording protocols used light, dark-light-dark programs, and acoustic-vibrational tapping as stimuli. One day prior to the recording, zebrafish larvae were carefully transferred into each well of a 24-well plate, with 1 ml Danieau’s medium per well. For locomotor recording, each plate was placed in the DanioVision system acclimated at 28 °C and the larvae were allowed to habituate to the new environment for 15 min. After this period, locomotor activity was continuously tracked for 1 h under standard light conditions. Next, the tapping program started with ten tap stimuli (intensity setting: 6) at intervals of 30 s. After a recovery period of 5 min, the light-dark program was initiated with alternating intervals of 15 min. All recordings were performed between 2 and 6 pm. Tracking experiments were performed in independent triplicates (with at least 24 larvae per genotype per replicate). Technical outliers (i.e., larvae not detected by the system) were removed from the quantification. Statistical analysis was performed using the GraphPad Prism analysis software (v10.2.1).

### Oil-Red O staining

11 and 13 dpf larvae were euthanized in 0.04% MS222 (buffered to pH∼7.5) and fixed in 4% paraformaldehyde overnight at 4 °C. The next day, fixed larvae were washed in 1X DPBS (no calcium, no magnesium, Gibco) and stained in 0.3% Oil-Red O (Sigma-Aldrich, #O1391) as described in Takashima et al. (2021).

### Histology and immunohistochemistry

The eyes and the liver of adult zebrafish were harvested and fixed as described above. Prior to usage, the adult eyes and livers were washed in 1X PBS (Gibco) and pre-embedded in 15% sucrose solution (in 1X PBS) for 1 h at room temperature for cryoprotection, and then in 30% sucrose solution (in 1X PBS) overnight at 4 °C. The organs were then embedded and frozen in Optimal Cutting Temperature (OCT) (VWR, Radnor, PA, USA) compound in specific cryomolds containing the orientation information (VWR, #720-0821).

Retinal cryo-sections (5 μm) (Leica CM1800 Cryostat) were blocked (0.1% Triton, 10% BSA in 1X PBS) for 1 h at room temperature, washed in 1X PBS, incubated at 4 °C overnight with primary antibody in incubation buffer (0.1% Triton in 1X PBS), washed, incubated at room temperature (RT) for 90 min with secondary antibody, washed, incubated 15 min with DAPI and washed again. Primary and secondary antibodies are described in Supplementary Data 1. Coverslips were mounted using ProLong Gold antifade reagent (Invitrogen, Carlsbad, CA, USA) and retinas were visualized using a Zeiss LSM780 confocal microscope and Zeiss Zen Black software version 2012. Retinal layer measurements were performed using ImageJ (RRID:SCR_003070). For each microscopy image, the thickness of the retinal layer was quantified at 20× magnification. Images were analyzed using ImageJ software. The thickness of the outer nuclear layer (ONL) and inner nuclear layer (INL) was measured at three equidistant points along the retinal section, spanning from the dorsal to the ventral edge. At each point, a straight line was drawn perpendicular to the retinal layers, and the thickness was recorded using the “Measure” function. The mean and standard deviation of ONL and INL thickness were calculated for each group, and data were presented as the average thickness per layer across all measured points. Glutamine synthetase (GS) intensity and retinal layer thickness based on GS-labeled cells were quantified using a MATLAB script (RRID:SCR_001622) designed for image analysis (version R2024a). The script measures the fluorescence intensity and calculates the length of glutamine synthetase-positive regions within the images. Similarly, cone density was quantified as the number of cones per unit area using the same MATLAB script. These automated analyses ensured consistent and reproducible quantifications across all samples.

Fixed livers (4% PFA at 4 °C overnight) were dehydrated with increasing concentration of ethanol (30%–100%) for tissue preservation. Livers were placed in cassettes (Epredia, Fisher Scientific, #10-009-57) and fitted in an automated paraffinization machine (Leica ASP300S) for dehydration, clearing, and inclusion of paraffin into the organ. After embedding, the paraffin block was sectioned with a microtome (Leica RM2155) into 10 μm slices. The latter were dipped in water at 42 °C and placed on cover slides to adhere. Slices were deparaffinized with xylene and decreasing ethanol concentrations (100%–30%) for 5–10 min each. The slides were quickly washed and then incubated in Mayer’s Haematoxylin (Rowley Biochemical, #L-756-1A) for 15 min, followed by rinsing in warm water and washing for a minute. After that, slides were stained in eosin for one minute and dehydrated with increasing concentrations of ethanol and, lastly, xylene. The slices were mounted with a coverslip using Permount (Fisher Scientific, #SP15-100). Livers were visualized using a Leica DMI600 microscope with DFC345FX camera and LASX software (Richmond Hill, Canada). Quantification of peroxisomes in zebrafish liver sections was performed using ImageJ. Images were converted to 8-bit grayscale and subjected to Gaussian blur to reduce noise. Background was subtracted using the rolling ball algorithm (∼20 pixels). Manual thresholding was applied to highlight peroxisomal puncta. The image was then converted to a binary mask, followed by erosion and watershed separation to distinguish closely apposed peroxisomes. Peroxisomes were quantified using the Analyze Particles tool, with size set to 10–200 pixels and circularity 0.10–1.00. The resulting count of peroxisomes was normalized to the number of DAPI-stained nuclei within each image. All quantitative analyses were performed on Average Intensity Projections to reflect peroxisomal content across the entire tissue sample. Data from multiple fields and biological replicates were compiled and analyzed in GraphPad Prism.

### Transcriptomics analysis

Larvae at 11 dpf were euthanized by rapid cooling (2–4 °C) and pools of 10 larvae collected in 0.5 ml Precellys tubes. Total RNA was extracted using Trizol as previously described in Heins-Marroquin et al. (2024) and RNA sequencing was performed by the Beijing Genomics Institute (BGI) using DNBSEQ™ sequencing technology platforms (Drmanac et al., 2010). The raw RNA-seq fastq files were quality-checked using the FastQC (RRID:SCR_014583) software (Babraham Institute Bioinformatics Group. FastQC, v0.12.0, 2023), and the data was pre-processed using the software package Rsubread (R Core Team, 2018; Liao et al., 2019). Gene-level differential expression analysis was conducted in the R statistical programming software (R Core Team, 2018) using the “DESeq2” (RRID:SCR_015687) package (Love et al., 2014) and filtering out genes with low expression counts using the filterByExpr-function with default parameters from the package edgeR (Robinson et al., 2010). *P*-value significance scores for differential expression were computed using the Wald test, and were adjusted for multiple hypothesis testing according to the Benjamini and Hochberg method (Benjamini and Hochberg, 1995) Pathway enrichment analyses were conducted with the clusterProfiler R package (RRID:SCR_016884, v3.16.0) (Yu et al., 2012) using Fisher’s Exact Test and the gene-level differential expression analysis results obtained with DESeq2 as input. The process network enrichment was visualized in a dot plot using React JS (v19). The pathway over-representation analysis statistics, including false-discovery rate (FDR) scores according to the method by Benjamini and Hochberg (Benjamini and Hochberg, 1995), were determined for gene sets from the Gene Ontology database using gene annotations specific to zebrafish, obtained from the Zebrafish Information Network (ZFIN) database (Sprague et al., 2003).

### Quantitative real-time PCR (qPCR)

Ten-fold diluted cDNA samples (2 μl) were added to a mixture (10 μl total volume) containing iQ SYBR Green Supermix (5 μl; Bio-Rad) and the primers of interest (Supplementary Data 1) at a final concentration of 0.25 μM. Reactions were run in a 384-well plate using a LightCycler^®^ 480 system (Roche). Expression levels were calculated relative to WT samples using the 2^–ΔΔCt^ method (Livak and Schmittgen, 2001). *Rpl13*α was used as a reference gene for normalization.

## Results

### Generation of a *pex1*-deficient zebrafish model showing reduced viability, impaired peroxisome biogenesis, and related metabolic perturbations

**FIGURE 1:**
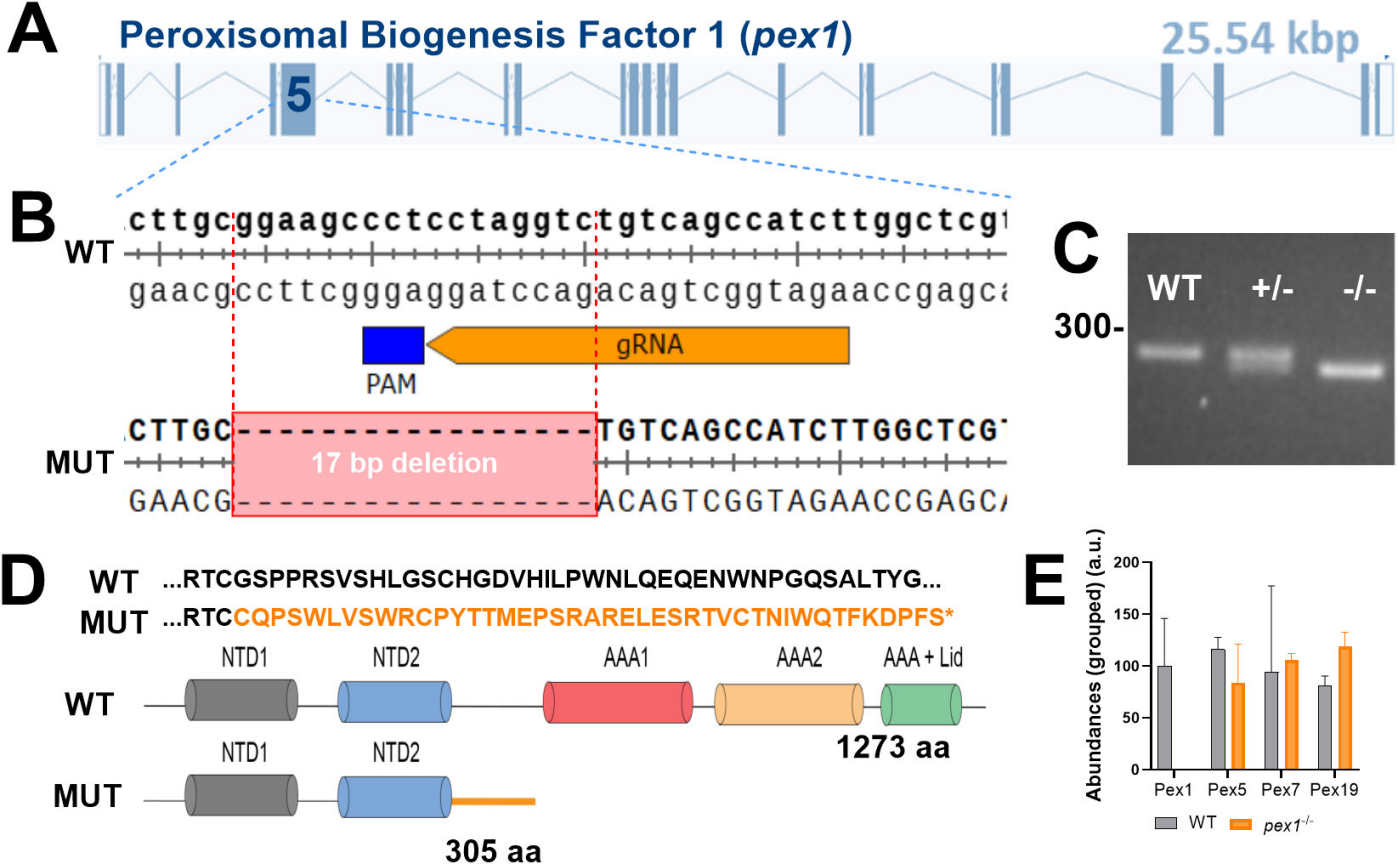
Generation of a *pex1* zebrafish model by CRISPR/Cas9. **(A,B)** Targeting CRISPR sites in exon 5 of the zebrafish *pex1* gene induced a 17 bp-deletion leading to a premature stop codon (MUT). **(C)** PCR on genomic DNA allows genotyping: wild-type (WT, 290 bp), heterozygous, and homozygous mutant (273 bp). **(D)** Amino-acid sequences of wildtype (WT, 1273 aa) versus mutated Pex1 protein (MUT, 305 aa), which is predicted to lack the AAA-ATPase domains essential for Pex1 function. The altered amino acid sequence resulting from the frameshift mutation is indicated in orange. **(E)** Proteomics-based analysis of Pex protein levels in livers from 7-month-old WT and *pex1*^–/–^ zebrafish (data are means ± % CV for *n* = 3 biological replicates).

The zebrafish genome encodes only one ortholog candidate of the human PEX1 protein (NP_000457.1). The putative zebrafish Pex1 protein (NP_001164306.1) comprises 1237 amino acids and presents 48% and 64% sequence identity and similarity to its human counterpart, respectively. Multiple sequence alignment of peroxisome biogenesis factor 1 proteins from human, mouse, zebrafish, and Drosophila confirmed high conservation of the two N-terminal domains (NTDs) and the two catalytic AAA-ATPase domains, supporting evolutionary conservation of protein function (Supplementary Figure 1). We generated a zebrafish *pex1*-deficient line using CRISPR/Cas9, targeting the fifth and longest exon of the gene (Figure 1A). This line (institutional code *pex1*^lux4^, following Zebrafish Information Network guidelines) carries a 17 bp deletion in the *pex1* gene, causing a frameshift and a premature stop codon in both genomic DNA and mRNA (Figures 1B, C). The predicted truncated protein (305 amino acids) lacks the C-terminal AAA-ATPase domains essential for Pex1 function (Figure 1D). Because antibodies for zebrafish Pex1 are unavailable, and we found that antibodies against human PEX1 did not cross-react, the absence of Pex1 protein could not be verified by immunoblotting. However, proteomic analysis confirmed the complete absence of the Pex1 protein in the *pex1*^–/–^ samples, while other Pex proteins were detected, validating the successful knockout of Pex1 in our zebrafish model (Figure 1E and Supplementary Data 1).

**FIGURE 2:**
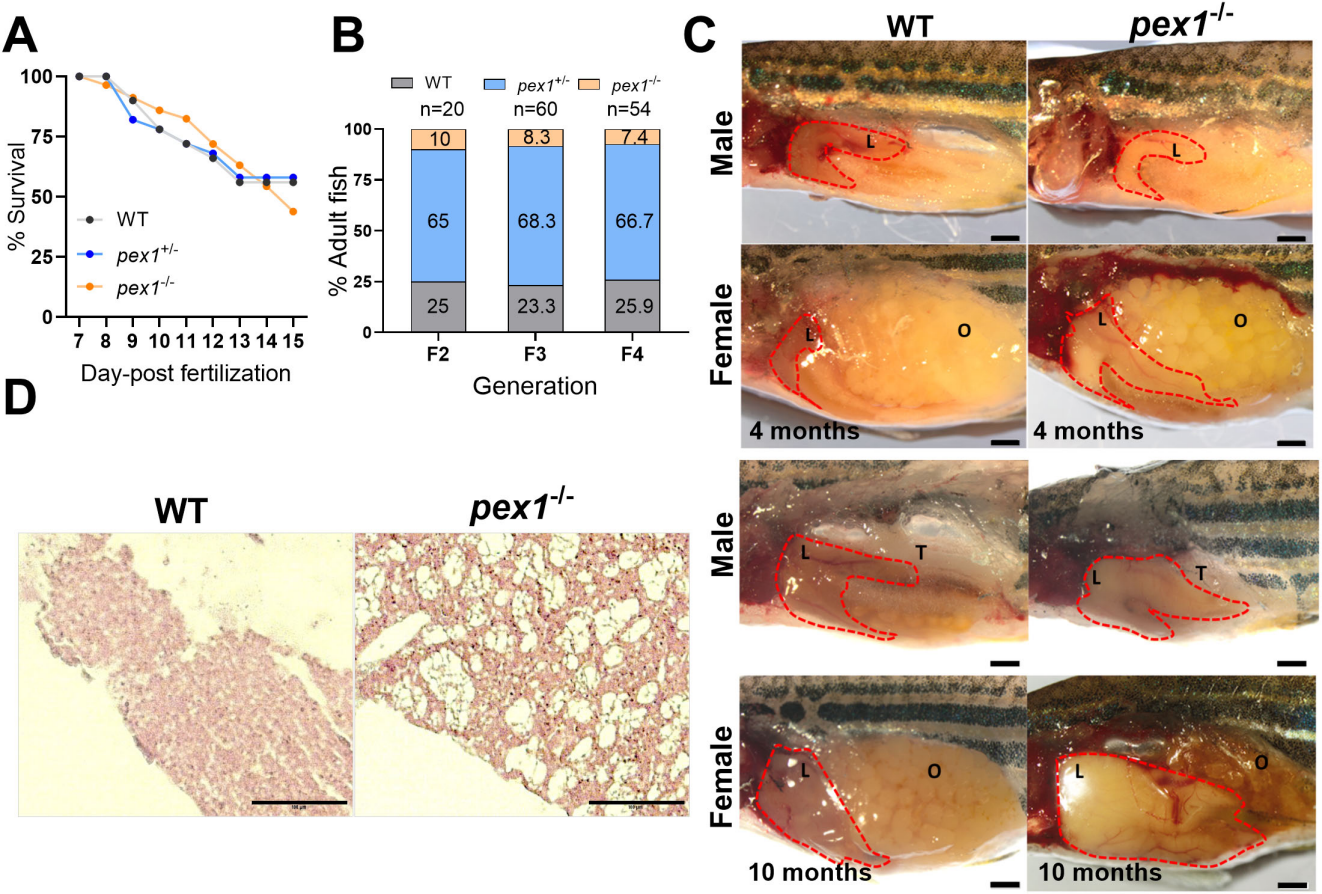
Survival and phenotypic characterization of the *pex1* mutant zebrafish line. **(A)** Survival curves up to 15 days post-fertilization (dpf) (*n* = 50). **(B)** Percentage of wild-type (WT), heterozygous (*pex1*^+/–^), and homozygous (*pex1*^–/–^) mutant offspring reaching adulthood from F2 to F4 generations. **(C)** For organ morphology characterization, ten and five animals per genotype were dissected at 4 and 10 months, respectively. Representative post-mortem images of WT and *pex1*^–/–^ female and male fish showing hepatic steatosis already at 4 months. Example of degenerated ovaries [O] and enlarged fatty liver in a 10-month-old female. Testis are indicated by a [T] in 10-month-old males. **(D)** H&E staining of liver sections from 7-month-old female fish revealed vacuolization in *pex1*^–/–^ samples (the images are representative of 2 biological replicates). Scale bars represent 100 μm in **(C,D)**.

To characterize this newly generated line and to determine whether *pex1* deficiency is lethal in zebrafish, F2 heterozygous (*pex1*^+/–^) fish were incrossed to obtain F3 offspring, yielding an expected 25% homozygous mutant (*pex1*^–/–^) progeny. A comparable survival rate was observed between *pex1*^–/–^, *pex1*^+/–^ and WT siblings in the F3 offspring until 15 dpf, suggesting that *pex1* is not essential at early developmental stages (Figure 2A). We suspect, however, that Pex1 function plays a critical role during juvenile stages, as *pex1*^–/–^ fish represented only 7%–10% of the population (instead of 25%) by 90 dpf (Figure 2B). Notably, these *pex1* knockout adult “survivors” did not show any overt phenotype. However, their lifespan was significantly reduced, and spontaneous death was observed between 6 and 10 months. A humane endpoint was applied to all fish (WT and *pex1*^–/–^) once spontaneous deaths occurred in the *pex1*^–/–^ population for three consecutive days. In addition, we suspect that *pex1*^–/–^ animals are infertile, as repeated crossing attempts failed to produce any viable offspring. The *pex1*^–/–^ females seemed to have functional ovaries producing eggs, which were, however, not released via natural spawning, eventually leading to gonad degeneration (Figure 2C). No significant differences were observed in body length between 7-month-old *pex1*^–/–^ and WT fish (Supplementary Figure 2A). Interestingly, both *pex1*^–/–^ males and females had developed fatty livers by 4 months of age. These livers typically presented a dense and yellowish appearance and, in some cases, were markedly enlarged, distinguishing them clearly from the reddish livers of WT siblings (Figure 2C). Histological analysis of liver sections confirmed massive macrosteatosis in 7-month-old *pex1*^–/–^ fish (Figure 2D).

**FIGURE 3:**
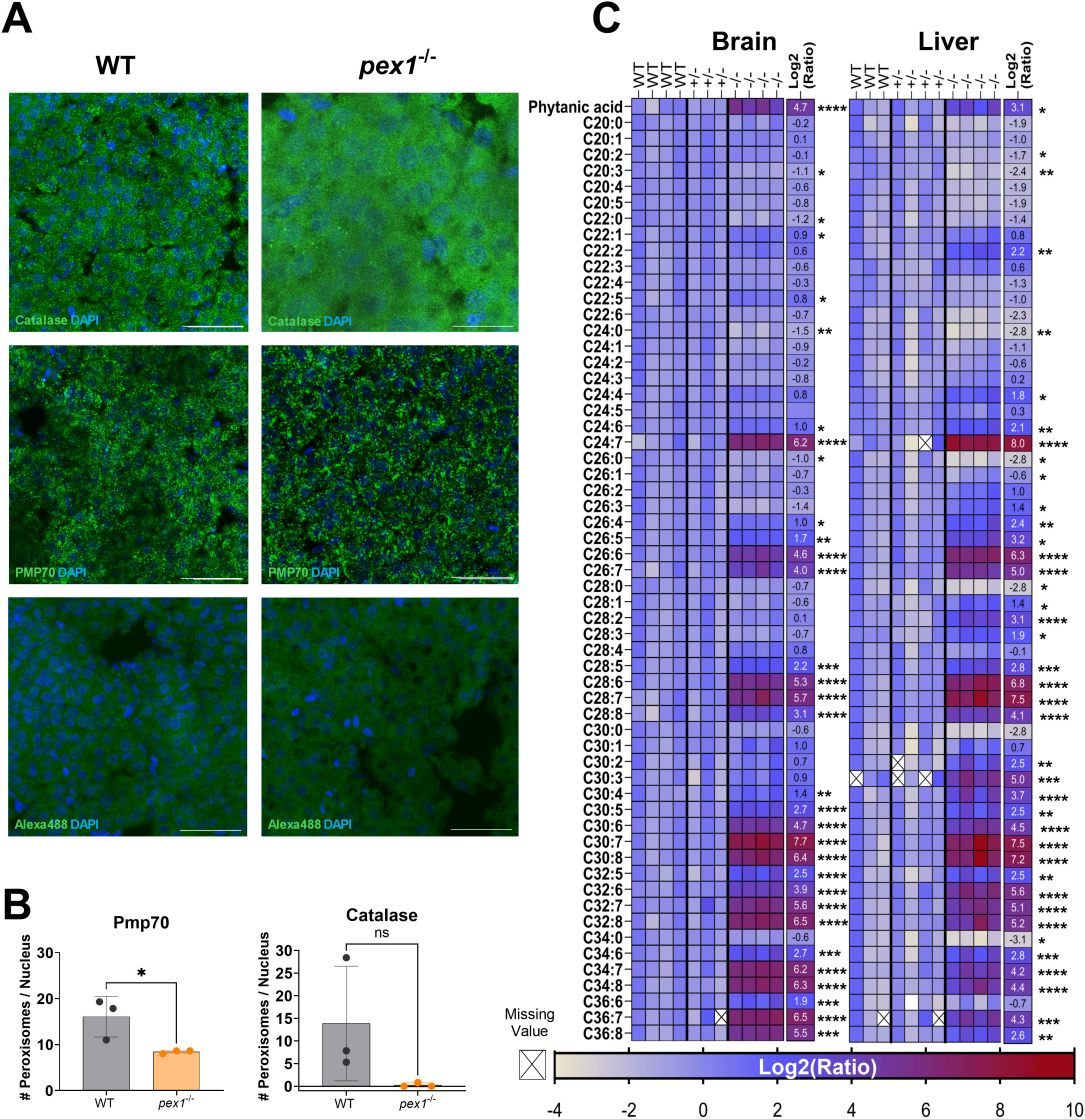
Peroxisome biogenesis is compromised in adult *pex1* zebrafish. **(A)** Confocal images of 5 μm liver sections from 7-month-old wild-type (WT) and *pex1*^–/–^ fish stained for catalase (peroxisomal matrix marker) or PMP70 (peroxisomal membrane marker). WT hepatocytes display numerous punctate peroxisomes, whereas *pex1*^–/–^ cells show a more diffuse catalase staining and fewer PMP70-positive puncta. Representative images were generated using Maximum Intensity Projection of the entire z-stack to enhance peroxisomal contrast. Secondary-antibody-only controls (Alexa488) verify staining specificity. Nuclei were counterstained with DAPI (cyan). Scale bars, 2 μm. **(B)** Quantification of peroxisome counts using Average Intensity Projection data. Bars represent means ± SD from three biological replicates (one section per fish; *n* = 3). Unpaired, two-tailed Student’s *t*-test: *p < 0.05; ns, not significant. **(C)** Heat-map of indicated fatty acid profiles measured by LC-MS in brain and liver of individual 7-month-old WT, *pex1*^+/–^, and *pex1*^–/–^ fish. The numerical values shown on the right correspond to KO/WT log_2_ ratios and represent means of 3–4 biological replicates. Crosses on white background indicate missing values. Multiple unpaired Student’s *t*-test: *p < 0.033, **p < 0.0021, ***p < 0.0002, ****p < 0.0001.

To assess peroxisome biogenesis in our *pex1* mutant model, we immunostained for two complementary peroxisome markers in 7-month-old zebrafish livers: catalase, a matrix enzyme that relies on the PEX5-mediated PTS1 import pathway, and PMP70 (ABCD3), an integral membrane protein that is inserted into peroxisomes via the PEX19/PEX3/PEX16 route and therefore does not require PEX5 (Fang et al., 2004; Okumoto et al., 2020). As expected, catalase staining in WT hepatocytes appeared as fine, punctate foci typical of peroxisomes. In *pex1*^–/–^ hepatocytes, however, the catalase signal was more diffuse throughout the cytosol, indicating a failure of matrix-protein import (Figures 3A, B). PMP70 still formed puncta in mutant tissue, confirming that membrane proteins can be incorporated independently of Pex5, yet the number of these puncta was markedly reduced compared with WT. This reduction points to a lower peroxisome abundance rather than a complete loss of the organelle.

To probe peroxisomal metabolism in the *pex1* mutant model, we measured fatty acids in liver and brain extracts of ≈7-month-old fish by LC-MS. Heterozygous and WT siblings showed similar levels across all analytes, confirming that a single wild-type *pex1* allele sustains normal peroxisomal function. In contrast, *pex1*^–/–^ tissues accumulated high levels of phytanic acid, whose α-oxidation is exclusively peroxisomal, and polyunsaturated VLCFAs (C24 and C26) and ultra-long-chain fatty acids (ULCFAs, ≥ C27), reaching log_2_ fold changes > 8. These lipid signatures demonstrate impaired peroxisomal α- and β-oxidation, corroborating the loss of functional peroxisomes in *pex1*^–/–^ fish (Figure 3C; Supplementary Figures 2B–D, 3 and Supplementary Data 1).

Interestingly, unlike in ZSD patients (Takashima et al., 2017), the brains of *pex1*^–/–^ zebrafish showed significantly lower levels of saturated C22 and C24 (Supplementary Figure 2B), and overall saturated FA levels were consistently decreased in *pex1*^–/–^ brain and liver samples, in stark contrast to the increased PUFA levels (Supplementary Figures 2B–D, 3). A notable exception among the PUFAs was docosahexaenoic acid (DHA, C22:6n-3), whose levels were decreased in the brain and in the liver of *pex1*^–/–^ fish compared to WT (Supplementary Figures 2B, 3). Although this difference did not reach statistical significance, the significantly increased levels of C24:6 (peroxisomal DHA precursor) are consistent with metabolically impaired peroxisomes. The more moderate than expected decrease in DHA levels [Zellweger syndrome patients show strongly decreased tissue DHA levels (Martínez, 1990)] may be explained by an alternative pathway for DHA biosynthesis reported in teleost fish, known as the “Δ4 pathway” (Oboh et al., 2017), or by dietary factors, potentially compensating, at least in part, for deficient peroxisomal DHA biosynthesis. To investigate the latter possibility, we analyzed the fatty acid content of the fish food (Supplementary Figure 4). The SDS100 dry food formulated for feeding in early developmental stages (5 dpf to max. 30 dpf) showed in general higher levels of FAs and DHA compared to SDS400 (dry food for adult maintenance) and live feed. Among the live feed, rotifers, intended for feeding young larvae, contained ten times more DHA than *Artemia nauplii*, the live feed for juvenile larvae and adult fish. Overall, this fish feed is relatively rich in DHA. For instance, SDS400 and rotifers contain about 10 mg of DHA per gram (≈1% w/w), a concentration in the same order of magnitude as that found in the most DHA-rich human foods, i.e., cold-water oily fish such as mackerel, herring, and salmon (Mozaffarian and Rimm, 2006). This suggests that the zebrafish nutrition may partially compensate for the lack of DHA biosynthesis in the *pex1*^–/–^ larvae and adults. Except for the more moderate than expected decrease in DHA levels, our observations in adult *pex1*^–/–^ zebrafish suggest that Pex1 plays an important role for proper peroxisomal function in zebrafish.

### Lipid homeostasis is perturbed in *pex1*^–/–^ zebrafish larvae

**FIGURE 4:**
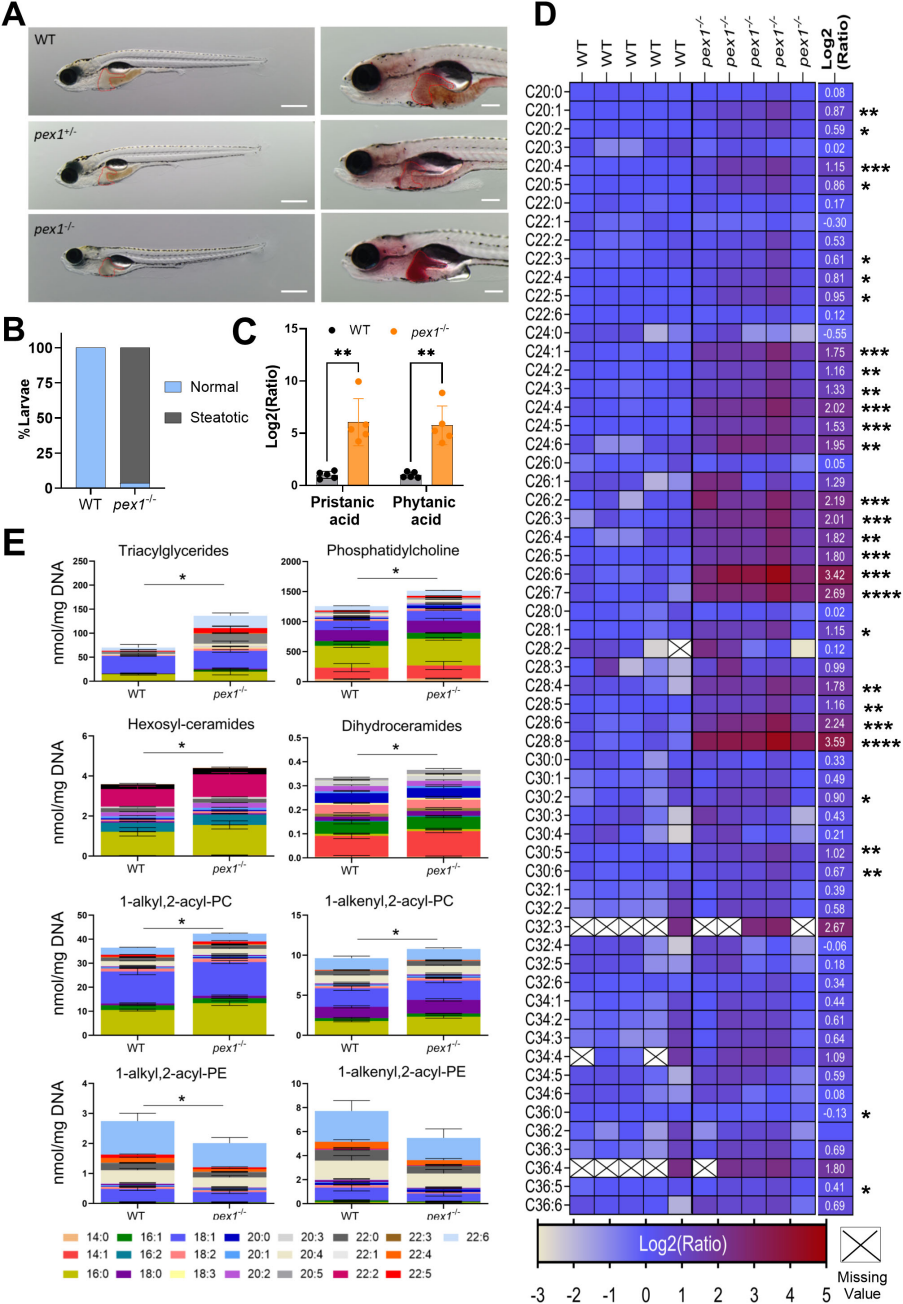
Liver steatosis and lipid dyshomeostasis manifest during early development in *pex1* mutant larvae. **(A)** Brightfield (left) and Oil-Red O (ORO) staining (right) of 13 dpf zebrafish larvae. Robust neutral-lipid accumulation is confined to the liver of *pex1*^–/–^ larvae compared with wild-type (WT) and heterozygous siblings. Scale bar, 1 mm. **(B)** Incidence of ORO-positive (steatotic) livers at 13 dpf; *n* = 20 larvae per genotype. **(C)** Log_2_ fold change of pristanic and phytanic acid in *pex1*^–/–^ versus WT larvae at 13 dpf measured by LC-MS in whole-larva extracts. Bars represent means ± SDs of five independent replicates, each replicate being a pool of five larvae (*n* = 5). **(D)** Heatmap of fatty acid (FA) species profiled by LC-MS. Columns represent biological replicates (pools of five larvae at 11 dpf); rows represent individual FAs. The numerical values on the right correspond to KO/WT log_2_ ratios and statistical significance was assessed with multiple unpaired Student’s *t*-test: *p < 0.033; **p < 0.0021, ***p < 0.0002, ****p < 0.0001. Crosses on white background indicate missing values. **(E)** Stacked bar plots of the FA composition of the indicated lipid classes determined by targeted lipidomics at 13 dpf. Data are means ± SDs of five biological replicates (pools of 15 larvae) normalized to DNA content. Statistical significance in panels **C,E** was assessed with an unpaired Welch’s *t*-test (WT vs. *pex1*^–/–^). *p < 0.033; **p < 0.0021, ***p < 0.0002, ****p < 0.0001.

To identify the earliest phenotypes in our *pex1*^–/–^ zebrafish model, homozygous mutant larvae were visually evaluated under brightfield light microscopy. They showed completely normal development until 7 dpf, with no significant difference in body length when compared to WT siblings. However, 96% of *pex1*^–/–^ larvae at 11 dpf showed darkening of the liver, and hepatic steatosis was confirmed using oil red (ORO) staining at 13 dpf (Figures 4A, B). Accumulation of polyunsaturated VLCFAs (C24–C30) and methyl-branched fatty acids was confirmed in 11 dpf *pex1*^–/–^ larvae using LC-MS (Figures 4C, D and Supplementary Data 1). The most prominently elevated FAs were C26:6, C26:7, C28:8, phytanic acid, and pristanic acid, which showed a log_2_ fold change higher than 2 in *pex1*^–/–^ larvae compared to the WT siblings.

To further characterize alterations in lipid homeostasis, we performed a targeted lipidomics analysis of lipid species with C14–C22 acyl chains (Supplementary Data 1). Our analysis revealed that, among the lipids analyzed, triacylglycerides (TGs) were the main altered lipid class in *pex1*^–/–^ larvae (136.4 ± 53.40 nmol/mg DNA), with about 2 times higher total TG levels than in WT larvae (70.2 ± 20.33 nmol/mg DNA) (Figure 4E). There was a significant increase in polyunsaturated C20 and C22 FAs, including C22:6, and a lower abundance of shorter, saturated and mono-unsaturated FAs such as C16:0 and C18:1, in the composition of TGs, in *pex1*^–/–^ larvae compared to WT siblings (Supplementary Figures 5A, B).

Besides TGs, there was a slight but significant increase in hexosylceramides, dihydroceramides, phosphatidylcholine (PC), and phosphatidylcholine plasmalogen (PC-PL) in the *pex1*^–/–^ larvae; however, no major alterations in FA composition were observed in these lipid classes (Figure 4E). Interestingly, a consistent decrease in phosphatidylethanolamine (PE) and phosphatidylethanolamine plasmalogen (PE-PL) was observed in the *pex1*^–/–^ larvae, with only 1-alkyl,2-acylphosphatidylethanolamine being significantly lower (Figure 4E). For the remaining analyzed lipids, including diacylglycerides (DG), phosphatidylinositols (PI), phosphatidylserines (PS), phosphatidylglycerols (PG), cholesterol esters (CE), ceramides (CER), lactosyl-ceramides (Lac-Cer), lysophosphatidylethanolamines (LPE), and lysophosphatidylcholines (LPC), no significant changes were found in the *pex1*^–/–^ larvae *versus* WT siblings (Supplementary Figure 5C).

### Transcriptomics analysis indicates increased ER stress in *pex1*-deficient larvae

**FIGURE 5:**
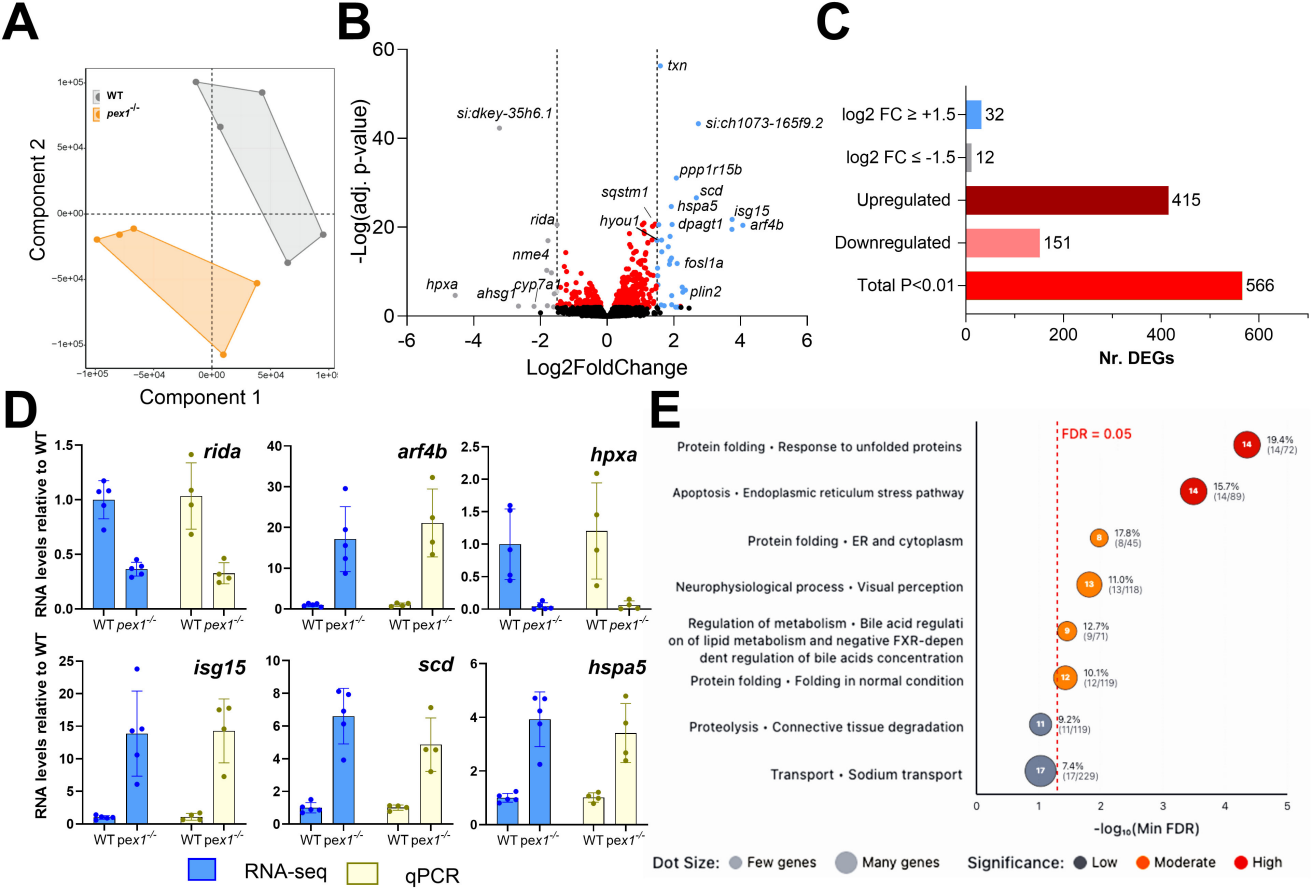
Transcriptomics analysis of *pex1* larvae indicates ER stress, alteration of neurological and visual processes, and dysregulation of bile acid metabolism. **(A)** Partial Least Squares Discriminant Analysis (PLS-DA) of gene transcripts of 11 dpf WT and *pex1*^–/–^ larvae. Each dot represents a single biological replicate of a pool of ten WT (gray) or *pex1*^–/–^ (orange) larvae. **(B)** Volcano plot showing significantly altered genes (adj. *p*-value < 0.01) in red, strongly downregulated genes (log2FC ≤ –1.5) in gray, and strongly upregulated genes (log2FC ≥ + 1.5) in blue. **(C)** Summary bar plot of the number of DEGs identified by the transcriptomics analysis. **(D)** qPCR validation of selected DEGs identified by RNAseq. All expression levels in *pex1*^–/–^ larvae are shown relative to WT levels. **(E)** Dot plot showing cellular process networks significantly enriched in differentially expressed genes. Dot position along the *x*-axis represents -log10(minFDR) values, with dots further right indicating higher statistical significance as measured by the false-discovery rate (FDR). Dot size reflects the number of genes found in each process, and color intensity indicates significance level. Processes related to protein folding and endoplasmic reticulum stress pathways show the strongest enrichment signals.

We next performed bulk RNA sequencing on *pex1*^–/–^ larvae and WT siblings at 11 dpf. Partial least squares discriminant analysis (PLS-DA) separated the samples by genotype along the first component, indicating that the *pex1* mutation produces a distinct transcriptomic profile (Figure 5A). Differential gene expression analysis revealed 566 differentially expressed genes (DEGs) (p-adjust < 0.01), with 415 upregulated and 151 downregulated DEGs in *pex1*^–/–^ *versus* WT larvae (Figures 5B, C and Supplementary Data 1). Six DEGs (*rida*, *arf4b*, *hpxa*, *isg15*, *scd*, and *hspa5*) were validated by qPCR, corroborating the transcriptomic findings (Figure 5D). Among the DEGs, 12 downregulated and 32 upregulated genes displayed a log_2_ fold change of more than 1.5. Among these, *arf4b*, *isg15*, *scd*, *ppp1r15b*, *fosl1a*, and *plin2* were the most upregulated, while the liver-specific genes *hpxa*, *ahsg1*, and *cyp7a1* were the most downregulated genes in the *pex1*^–/–^ larvae. These data implicate the liver as an early, and possibly initiating, site of pathology in this model.

To identify altered pathways potentially relevant for Zellweger syndrome, DEGs were mapped to their human orthologs according to the HUGO Gene Nomenclature Committee (HGNC). Pathway enrichment analysis was then performed using the Human Pathway Maps and Cellular Process Networks from the GeneGO database. The top-ranked pathways/processes (*p* < 0.05) included the endoplasmic reticulum (ER) stress response, neurophysiological process/visual perception, bile acid metabolism and negative FXR-dependent regulation of bile acid concentration, connective tissue degradation, and sodium transport (Figure 5E and Supplementary Data 1). Network visualization diagrams show upregulation of different branches of the ER stress pathway (Supplementary Figure 6), particularly downstream of the ATF-6 and IRE1 signaling pathways that activate pexophagy (*sqstm1*) and the protein folding response (*GRP78*, *DnaJB9*, *Endoplasmin*, *hspa5*). This indicates that peroxisomal dysfunction leads to ER stress and peroxisomal degradation. Visualization of the “neurophysiological process_visual perception” network highlighted downregulation of genes encoding especially light-sensitive receptors and alterations in their downstream pathways (Supplementary Figure 7). Interestingly, results showed a mild, non-significant decrease in all *pex* genes, suggesting that there is no transcriptional regulation of peroxisomal genes to compensate for Pex1 deficiency. The transcript levels of the *pex1* gene itself were also only decreased by about 25% in the *pex1*^–/–^ larvae (as confirmed by qPCR; not shown), indicating that the mutant allele (17 bp deletion) does not undergo nonsense-mediated mRNA decay.

**FIGURE 6:**
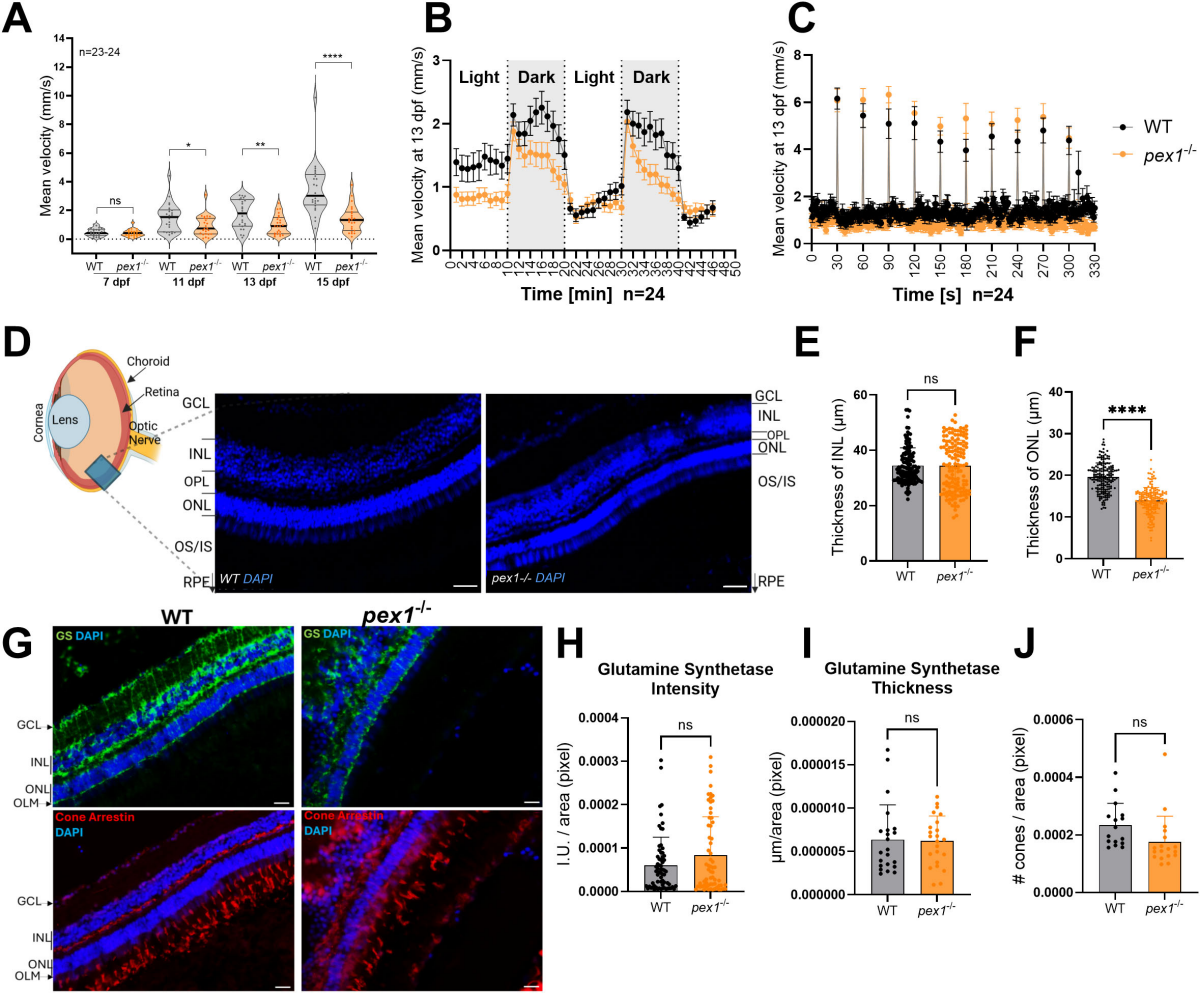
Pex1 deficiency leads to behavioral changes in larvae and perturbed retinal architecture in adult fish. **(A)** The total mean velocity of 7, 11, 13, and 15 dpf larvae was recorded under constant light conditions for 1 hr. Each dot represents the mean velocity of one larva (*n* = 23–24). **(B)** Behavioral profiles (1-min intervals) of 13 dpf larvae under alternating light/dark conditions in 10-min interval cycles. **(C)** Startle response profile (1-sec intervals) of 13 dpf larvae upon acoustic stimuli applied in 30-sec intervals. Each dot in **(B,C)** represents the mean velocity (± SEM) of 24 larvae recorded on two independent plates. Statistical significance in **(A)** was determined by unpaired *t*-test (*p < 0.033; **p < 0.0021, ****p < 0.0001). **(D)** Zebrafish eye representation showing DAPI counterstaining of nuclei (blue) across the retinal layers—GCL, INL, OPL, and ONL—in 7-month-old animals. Scale bars represent 10 μm. **(E,F)** Six measurements were taken from six images per eye, with three images on each side of the optic nerve. For each side, these three images were positioned equidistant from the optic nerve. Each dot represents an individual measurement. Values are presented as means ± SDs; statistical analysis was performed using an unpaired *t*-test (****p < 0.0001). **(G)** Glutamine synthetase (GS; green) and cone arrestin (red) immunostaining highlighting Müller glial and cone cells, respectively. Scale bars represent 5 μm. **(H,I)** Quantification of Müller cell fluorescence intensity and cell layer thickness (μm) normalized to Müller cell area (operationally defined as the area within the retinal section that shows positive GS labeling). Measurements were taken from three images lateral to the optic nerve on both left and right sides (6 images/eye). The Müller cells were delineated based on GS staining from the outer limiting membrane to the inner nuclear layer. Each dot represents a single measurement. **(J)** Normalized cone density (number of cones/area). Cones were automatically identified using a custom Matlab script, with manual correction for overlapping signals. Each dot represents one single image taken from three images lateral to the optic nerve, as described above. For **(H–J)**, means ± SDs are shown and statistical analysis was performed using an unpaired *t*-test (ns, non-significant). Three **(E,F)** or four **(H–J)** eyes from different animals were analyzed per genotype. GCL, ganglion cell layer; INL, inner nuclear layer; OPL, outer plexiform layer; ONL, outer nuclear layer; OS/IS, outer/inner segment; RPE, retinal pigment epithelium; GS, glutamine synthetase; OLM, outer limiting membrane; I.U., intensity units.

To assess potential neurological phenotypes, we analyzed the behavior of the larvae. First, we performed locomotion assays at different developmental stages and under normal light conditions. At 7 dpf, no significant differences were observed between the larvae of different genotypes (Figure 6A and Supplementary Figure 8A). However, starting at 11 dpf, *pex1*^–/–^ larvae showed a consistent decrease in movement, which became more evident at 13 and 15 dpf, indicating that their locomotion is progressively impaired in comparison to WT controls (Figure 6A and Supplementary Figure 8A). The difference in the locomotor behavior does not seem to be related to any obvious developmental defect, since *pex1*^–/–^ larvae show similar morphology and size compared to their WT siblings (Supplementary Figures 8B, C). To further evaluate locomotion-based behaviors, we performed startle response assays by delivering drastic light changes and tapping as stimuli. Similarly to the first assay, 7 dpf *pex1*^–/–^ and WT larvae showed no obvious differences in the expected behavioral response that is characterized by a transient period of hyperactivity as soon as there is a dark flash, followed by a progressive return to basal activity, which drops as soon as there is a light flash (Supplementary Figure 8D; Burgess and Granato, 2007). However, at later stages, *pex1*^–/–^ larvae displayed a shorter adaptation period to dark conditions than their WT sibling larvae, and the mutants did not show the sudden drop-in locomotor activity upon the light flash (Figure 6B). Interestingly, we did not observe a difference in the startle response upon tapping, which is an acoustic-vibrational stimulus generated by a built-in solenoid in the behavioral chamber and capable of inducing fast motor escape responses (Figure 6C and Supplementary Figure 8E). Such responses are mediated by Mauthner cells and associated neural pathways in the hindbrain (Liu and Fetcho, 1999).

The observations in the behavioral assays together with the transcriptomics data support the idea that visual perception pathways might be affected in the *pex1*^–/–^ larvae. Considering that ZSD patients develop retinal degeneration and glaucoma (Yergeau et al., 2023), we sought to assess whether similar hallmarks are present in our *pex1*^–/–^ zebrafish model. Eyes of 7-month-old *pex1*^–/–^ fish were cryosectioned and stained with DAPI. The inner nuclear layer (INL) did not show a significant difference in thickness between the *pex1*^–/–^ and WT samples (34.43 ± 1.82 μm and 34.49 ± 1.94 μm) (Figures 6D, E). However, the thickness of the outer nuclear layer (ONL) was significantly reduced in the *pex1*^–/–^ samples compared to the WT controls (14.01 ± 4.89 μm and 19.57 ± 6.23 μm, respectively) (Figures 6D, F). To further investigate changes in these layers, we performed immunohistochemistry on specific cell types. These analyses did not show statistically significant alterations in the overall number and structural organization (thickness) of Müller glial cells, spanning from the outer limiting membrane (OLM) to the ganglion cell layer (GCL) based on glutamine synthetase (GS) staining (Figures 6G–I). On the other hand, cone arrestin staining revealed a disorganized cone outer segment and a trend toward fewer cone cells compared to WT (Figures 6G, J). However, since the reduction in cone cell count was not statistically significant, the substantial thinning of the ONL suggests greater rod loss, leading to significant photoreceptor degeneration.

## Discussion

In this study, we established and characterized a zebrafish model of Zellweger spectrum disorders (ZSDs). We used CRISPR/Cas9 to introduce a 17-bp deletion in exon 5 of *pex1*, the zebrafish ortholog of the gene most commonly mutated in ZSD patients, *PEX1*. The deletion causes a frameshift that inserts a premature stop codon, leading to the absence of Pex1 protein and resulting in a marked reduction of functional peroxisomes. We characterized this novel ZSD model at both larval and adult stages and found key hallmarks of ZSDs, including pronounced accumulation of methyl-branched fatty acids and very long-chain polyunsaturated fatty acids (VLC-PUFAs) (Wanders et al., 1987; Kovacs et al., 2012; Wangler et al., 2018; Takashima et al., 2021).

A prominent phenotype in *pex1*^–/–^ larvae was hepatic steatosis, accompanied by increased TG levels. In the liver, TGs are stored within lipid droplets (LDs), neutral lipid-filled organelles originating from the ER and surrounded by a single phospholipid monolayer (Mathiowetz and Olzmann, 2024). Accordingly, our transcriptomic analyses revealed upregulation of *plin2*, a well-established LD marker, in *pex1*^–/–^ larvae. Beyond lipid storage, LDs have diverse roles in energy homeostasis, membrane and hormone biosynthesis, and they interact with several cellular compartments, including peroxisomes (Tsai et al., 2017). Notably, pharmacologically induced ER stress has been shown to promote LD formation, which may provide a mechanism to sequester and facilitate the removal of unfolded or misfolded proteins from the ER (Lee et al., 2012). In line with this, pathway-enrichment analysis of our RNA-seq data primarily highlighted upregulation of ER stress and unfolded-protein-response (UPR) modules in *pex1*^–/–^ larvae. This UPR activation may be triggered by physicochemical changes in the ER membrane that arise from the mutant lipid profile, namely the excess VLC-PUFAs and branched-chain fatty acids, possibly via ATF6 and/or other ER stress sensors. The reduced capacity for peroxisomal H_2_O_2_ detoxification may further aggravate the situation: elevated reactive oxygen species promote peroxidation of the VLC-PUFAs that accumulate in the ER, thereby amplifying the stress signal and sustaining UPR gene-network activation and elevating LD formation. This continuous cycle of oxidative damage and metabolic dysfunction likely represents a critical pathogenic mechanism underlying the progressive hepatocellular injury observed in peroxisomal biogenesis disorders. The sustained ER stress response, while initially adaptive, may eventually contribute to chronic inflammation and potential progression toward hepatic fibrosis (Valenzuela et al., 2012).

Another notable transcriptional feature in *pex1*^–/–^ larvae, likely linked to the ER stress response, was the strong induction of stearoyl-CoA desaturase (*scd*). Although our lipidomics dataset did not show obvious elevations of its classical products, palmitoleic (C16:1) and oleic (C18:1) acids, relative to their saturated precursors, upregulation of *scd* may still constitute a compensatory response to the complex lipid dyshomeostasis that characterizes the *pex1*^–/–^ model. Scd-derived monounsaturated fatty acids (MUFAs) are key determinants of membrane fluidity and serve as preferred substrates for TG and phospholipid synthesis (Paton and Ntambi, 2009); increasing their supply can help maintain membrane fluidity and facilitate the sequestration of surplus VLC-PUFAs and branched-chain acyl-CoAs into neutral lipid pools, thereby limiting lipotoxic stress. Interestingly, an unbiased chemical–genetic screen in a zebrafish *abcd1* mutant (X-linked adrenoleukodystrophy model) identified *scd* induction as a protective modifier, and pharmacological activation of Scd rescued abnormal motor behavior in that model (Raas et al., 2021). Induction of *scd* in *pex1*^–/–^ larvae may thus constitute an attempt to restore lipid homeostasis in response to markedly elevated VLC-PUFAs and branched-chain fatty acids.

Accumulation of VLC-PUFAs has also been reported in ZS patients (Poulos et al., 1988; Takashima et al., 2017). In contrast to ZS patients, however, saturated VLCFA levels, including C24:0 and C26:0, were not increased, but rather decreased in *pex1*^–/–^ larvae and adult tissues. Moreover, despite the expected impairment of ether lipid biosynthesis in peroxisome-deficient conditions, we did not observe a global reduction of plasmalogens in 13 dpf *pex1*^–/–^ larvae. Instead, our targeted lipidomics analyses revealed a moderate but significant shift in plasmalogen species composition, with increased levels of 1-alkyl-2-acyl-PC and 1-alkenyl-2-acyl-PC, and decreased levels of 1-alkyl-2-acyl-PE and 1-alkenyl-2-acyl-PE. Interestingly, transcriptomic analysis revealed a significant upregulation of phosphoethanolamine methyltransferase (ptm), which catalyzes the conversion of phosphoethanolamine to phosphocholine (Bobenchik et al., 2011), suggesting a redirection of plasmalogen metabolism toward PC synthesis at the expense of PE. Together, these data point to a complex regulatory adaptation of lipid metabolism in response to impaired peroxisomal function during early zebrafish development. A limitation of our targeted lipidomics approach is that it only covered acyl chains up to C22, preventing identification of the specific lipid species in which the accumulating VLC-PUFAs are incorporated. Future analyses using untargeted lipidomics with extended acyl chain coverage will be needed to identify the lipid species harboring VLC-PUFAs.

Another prediction from the transcriptomic data was perturbed visual perception in the *pex1*^–/–^ larvae, based on the downregulation of genes encoding light-sensitive receptors such as *rhodopsin*, *opn1sw2*, and *opn1sw1*. This molecular signature was corroborated by histopathological analysis of adult *pex1*^–/–^ fish, which revealed disorganization of the retinal layers, reminiscent of the progressive retinopathy observed in ZSD patients (Yergeau et al., 2023). In the adult *pex1*^–/–^ zebrafish, we observed a significant reduction of the thickness of the outer nuclear layer (ONL), consistent with the decline in rod and cone photoreceptor numbers observed in the Pex1-G844D mouse model (Argyriou et al., 2021). Accumulation of phytanic acid has been linked to retinal degeneration, brain and nerve damage (Mezzar et al., 2017); it could therefore be one of the causes for the retinal phenotype in the adult *pex1*^–/–^ fish and decreased locomotor behavior in the larvae. Moreover, studies in the Pex1-G844D mouse confirmed a correlation between the accumulation of VLCFAs and retinal degeneration, altogether showing the important role of functional peroxisomes to support vision health (Omri et al., 2025). In contrast to the early-lethal phenotypes reported for complete PEX loss in humans and mice (Baes et al., 1997; Hiebler et al., 2014), *pex1*^–/–^ zebrafish remain viable through embryonic, larval, and juvenile stages, although only about 10% survive to adulthood and the surviving adults are sterile. A similarly attenuated course was described for zebrafish lacking *pex2*, *pex13* or the VLCFA transporter *abcd1* (mutated in X-linked adrenoleukodystrophy), all of which reached adulthood yet showed VLCFA accumulation, fatty-liver pathology, locomotor impairment, and/or infertility (Strachan et al., 2017; Takashima et al., 2021; Demers et al., 2023). By contrast, *pex5* mutants died within the first month post-fertilization (Bhandari et al., 2023), indicating locus-specific differences in the threshold of peroxisome function required for early zebrafish development.

The comparatively mild disease course in *pex1*^–/–^ zebrafish potentially reflects a two-phase buffering system. During the first 24–48 h post-fertilization, the phenotype may be masked through maternal rescue, where embryos from heterozygous crosses inherit wild-type *pex1* transcripts, Pex1 protein, and intact peroxisomes (Despic et al., 2017). Indeed, as *pex1*^–/–^ zebrafish do not produce viable progenies, all *pex1*^–/–^ mutants in our study were obtained from *pex1*^+/–^ heterozygous crosses. Bhandari et al. (2023) showed that in *pex5* mutants, maternally transferred stores maintain punctate peroxisomes until 2 dpf and are exhausted by 5 dpf, after which the mutant phenotype emerges. In fact, offspring generated by homozygous *pex13*^–/–^ crosses died during the larval period (Demers et al., 2023), further supporting the idea that maternal mRNA indeed contributes to overcoming early lethality. A second mechanism for attenuating the ZSD phenotype may rely on the teleost-specific DHA biosynthesis pathway, which operates in zebrafish in addition to the peroxisome-dependent DHA synthesis pathway seen in mammals (Ferdinandusse et al., 2001). Zebrafish express an Fads2 Δ4-desaturase that converts C22:5n-3 to DHA (C22:6n-3) without requiring peroxisomal β-oxidation (Oboh et al., 2017). Furthermore, we found that standard laboratory diets used to raise the fish are rich in DHA, suggesting that dietary DHA may further contribute to the attenuation of the expected disease phenotypes. Consistent with this, instead of finding DHA depletion in the *pex1* mutant zebrafish, as would be expected from ZSD patients (Martínez, 1990), we found that DHA levels in triacylglycerols were actually double in 13 dpf *pex1*^–/–^ larvae compared to WT and that total DHA levels were only moderately reduced in adult mutants. Hence, it is possible that maternal effects postpone the onset of pathology, whereas the peroxisome-independent DHA pathway, and dietary lipids, attenuate its severity, allowing *pex1*^–/–^ (and *pex2*^–/–^, *pex13*^–/–^) fish to reach adulthood, in contrast to the perinatal lethality seen in mammalian *PEX* null models.

ZSDs are complex, multisystemic conditions in which the pleiotropic functions of peroxisomes are disrupted, perturbing numerous metabolic pathways across many organs (Klouwer et al., 2015). This makes it challenging to pinpoint the mechanisms that drive pathogenesis and to identify actionable therapeutic targets. Our data suggest that the *pex1*^–/–^ zebrafish model reproduces phenotypes that stem largely from the accumulation of VLC-PUFAs and methyl-branched FAs, providing a tractable system for dissecting the toxicity of these lipids. The absence of early lethality in this model further suggests that DHA deficiency may be a key contributor to the severe neonatal presentations of ZSD. In patients, one study reported that DHA ethyl-ester (DHA-EE) supplementation improved liver function, vision, muscle tone, and myelination (Martinez, 1996). However, a subsequent randomized, double-blind, placebo-controlled trial with microencapsulated powder containing DHA triglyceride (47% DHA) and arachidonic acid (AA) triglyceride (46% AA) showed that, although the oral treatment raised circulating DHA levels, it failed to improve visual function or growth (Paker et al., 2010). Stable-isotope tracing in zebrafish demonstrated that dietary DHA-EE is efficiently incorporated into phosphatidylcholine in the intestine, liver, and muscle, but is not detected in the brain, indicating that DHA-EE does not cross the blood–brain barrier and that cerebral DHA relies largely on *de novo* synthesis (Yoshinaga et al., 2022).

An additional factor that may underlie the comparatively mild phenotypes in our model is the species-specific composition of bile salts. Teleost fish synthesize mainly C27 bile alcohols and only minor amounts of C24 bile acids, whereas mammals rely almost exclusively on C24 bile acids (Hofmann et al., 2010; Wen et al., 2021). In mammals, the conversion of C27 to C24 species is completed by peroxisomal β-oxidation. C24 bile acids are potent ligands for the nuclear farnesoid X receptor (FXR); when FXR is activated it suppresses *CYP7A1*, the rate-limiting enzyme in bile-acid synthesis. Consequently, peroxisomal dysfunction lowers C24 bile-acid levels, disengages this negative-feedback loop, elevates *CYP7A1* expression and drives the accumulation of potentially cytotoxic C27 intermediates (Ferdinandusse et al., 2009; Anderson et al., 2021). The FXR signaling pathway is well conserved between zebrafish and mammals (Reschly et al., 2008; Wen et al., 2021), but, in zebrafish, both C24 bile acids and the predominant C27 bile alcohols can activate FXR (Makishima et al., 1999; Wen et al., 2021). Although we did not directly measure bile acids in our study, this provides a mechanistic explanation for the marked reduction in *cyp7a1* transcript levels observed in our *pex1*^–/–^ mutant despite the presumed low C24 bile acid levels.

**FIGURE 7:**
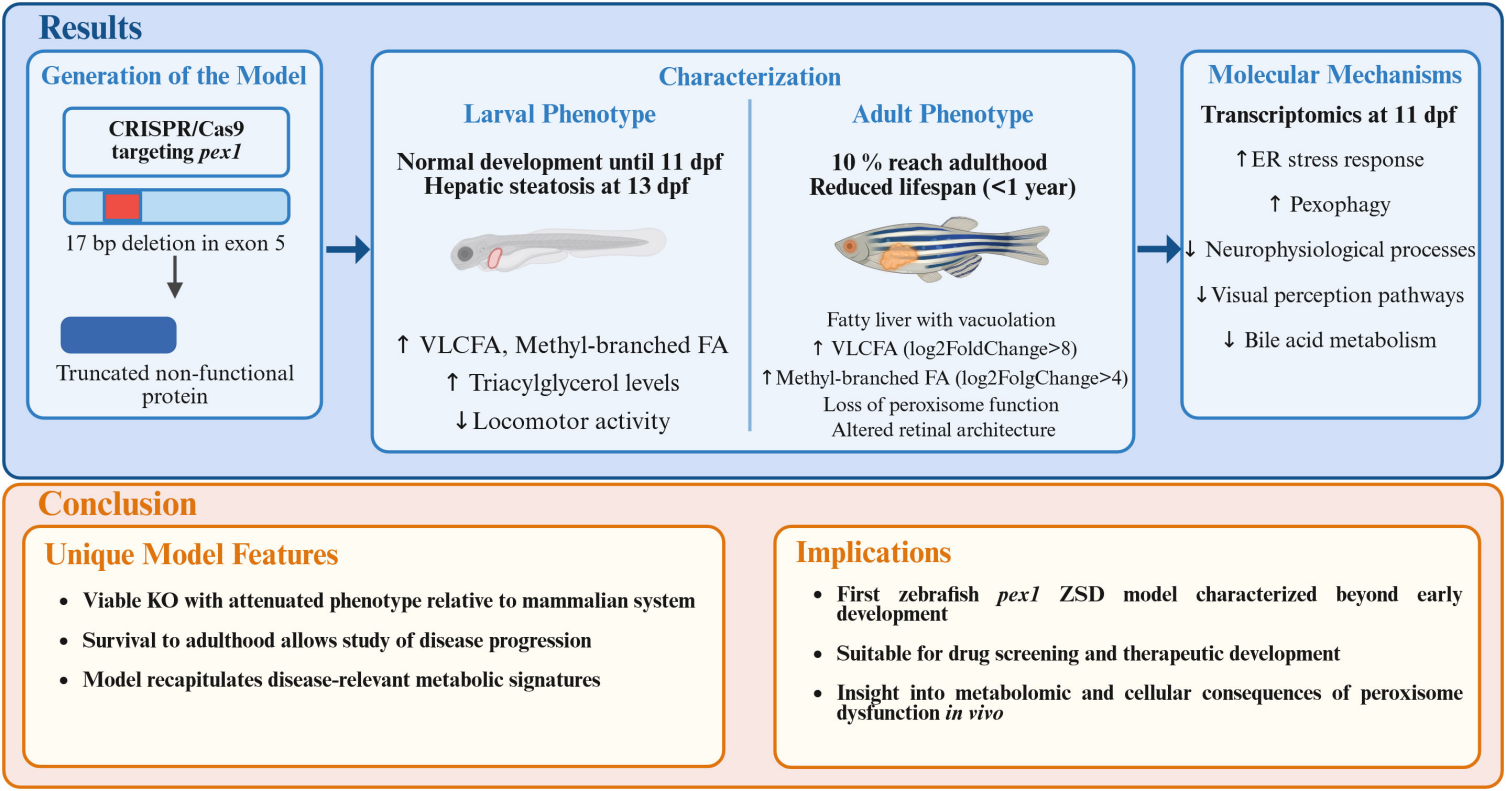
Schematic overview of the main phenotypic outcomes observed in the *pex1* ZSD zebrafish model, based on characterization through lipidomics, transcriptomics, histochemical immunostaining, and locomotor behavioral assays.

In summary, the *pex1*^–/–^ zebrafish fills an important gap between lethal mammalian *PEX*-null models and milder patient alleles: it reproduces major hallmarks of ZSD while remaining viable through larval and adult stages. Our data suggest a pathogenic cascade in which peroxisomal dysfunction drives VLC-PUFA and branched-chain FA overload, ER stress activation via membrane bilayer perturbation, compensatory Scd-mediated MUFA synthesis, LD formation, and progressive degeneration of the liver and of nervous system components. A summary overview of the mutagenesis strategy together with the principal phenotypic outcomes and their implications is presented in Figure 7.

All these phenotypes are quantifiable, robust, and detectable during early larval development, making them amenable to medium- and high-throughput screening. The model is particularly well-suited for drug discovery pipelines aimed at identifying small molecules or dietary interventions that restore lipid homeostasis, alleviate ER stress, or improve organ function. Using multi-parametric readouts—including fluorescent lipid reporters, live imaging of organ pathology, transcriptomic profiling, and metabolomic analysis—this system enables the assessment of therapeutic efficacy across molecular, cellular, and tissue levels. Together, these features position the *pex1*^–/–^ zebrafish as a versatile *in vivo* platform for screening mechanistically targeted therapies for ZSD and potentially other peroxisome-related disorders.

We note that a recent study investigated the role of Pex1 as a component of Notch signaling in cell fate determination and the differentiation of neural precursors in the p3 domain of zebrafish (Ryu et al., 2024). In this study, deletion of *pex1* in zebrafish led to an increased number of GABAergic KA” neurons and ectopic expression of secondary motor neurons, suggesting that Pex1 may have functions beyond peroxisomal biogenesis (Ryu et al., 2024). While our study primarily focused on modeling the metabolic and developmental consequences of *pex1* deficiency in the context of human ZSD, these findings highlight the broader utility of zebrafish for investigating both canonical and non-canonical roles of Pex1.

As discussed above, maternal *pex1* stores, a teleost-specific DHA-biosynthetic shortcut, and differential FXR regulation appear to mitigate the most critical aspects of the human disease, including early lethality and severe neurological manifestations. While these factors temper the disease phenotype, they also create a unique opportunity to explore modifier pathways that influence disease severity. In addition to drug discovery, this model can be used in future studies to further dissect ZSD pathomechanism (e.g., *in vivo* imaging of organ-selective lipid toxicity) and to clarify how disrupted peroxisomal metabolism perturbs cellular and organismal homeostasis.

## Data Availability

All data supporting the conclusions of this article are provided in the Supplementary material.

## Footnotes

1
http://chopchop.cbu.uib.no/
