# Supplementary material for: Pex1 loss-of-function in zebrafish is viable and recapitulates hallmarks of Zellweger spectrum disorders
Source: Front Mol Neurosci. 2025 Nov 5;18:1634536. doi: 10.3389/fnmol.2025.1634536 (PMC12626956; doi:10.3389/fnmol.2025.1634536)
Supplement: Supplementary file 1 [file Supplementary_file_1.docx]

**Pex1 Loss-of-Function in Zebrafish Is Viable and Recapitulates Hallmarks of Zellweger Spectrum Disorders**

**Ursula Heins-Marroquin^1†#^, Zlatan Hodzic^1†^, Beatriz Soares Carneiro da Silva^1^, Agnes Hendriks^1^, Floriane Gavotto^1^, Marc O. Warmoes^1^, Lisa Schlicker^1^, Samy Omri^2^, Christian Jäger^1^, Enrico Glaab^1^, Nancy E. Braverman^2,3^, Maria Lorena Cordero-Maldonado^1^, Carole L. Linster^1*^**

**^1^Luxembourg Centre for Systems Biomedicine, University of Luxembourg, L-4362 Esch-sur-Alzette, Luxembourg**

**^2^Child Health and Human Development Axis, Research Institute of the McGill University Health Centre, Montréal, Québec, Canada**

**^3^Division of Medical Genetics, Department of Pediatrics and Human Genetics, McGill University, Montreal, Québec, Canada.**

***Correspondence: Carole L. Linster,** [**carole.linster@uni.lu**](mailto:carole.linster@uni.lu)

**^†^These authors contributed equally to this work and share first authorship.**

**^#^Current affiliations: Department of Metabolic Diseases, Wilhelmina Children's Hospital, University Medical Center Utrecht, Lundlaan 6, 3584 EA, Utrecht, The Netherlands; Department of Genetics, University Medical Center Utrecht, Heidelberglaan 100, 3584 CX Utrecht, The Netherlands**

**Supplementary Material**

Supplementary Figures 1-8 with legends

Supplementary Data 1 (description)

**Supplementary Figure 1.** Evolutionary conservation of the PEX1 protein across species. Multiple sequence alignment of PEX1 orthologs from *Homo sapiens* (Hs, NP_000457.1), *Mus musculus* (Ms, NP_001280735.1), *Danio rerio* (Dr, NP_001164306.1) and *Drosophila melanogaster* (Dm, NP_652016.1). Shades of blue indicate sequence conservation, with dark blue representing highly conserved residues and white indicating no conservation. The domain organization of the type II AAA+ ATPase PEX1 is shown below the alignment, including the two N-terminal domains (NTD1 and NTD2), the two AAA+ ATPase domains (AAA1 and AAA2), and the AAA+Lid domain often found at the C-terminus of AAA+ domains. Corresponding regions are highlighted with matching colors in the alignment.

**Supplementary Figure 2.** Growth and brain VLCFA levels in adult *pex1* zebrafish. (A) Body length of a 7-month-old adult zebrafish. Each dot represents an individual fish; the horizontal line indicates the mean. Statistical significance was assessed using a two-tailed unpaired Student’s *t*-test (ns, not significant). (B–D) Very long-chain fatty acid (VLCFA) levels in brains of 7-month-old *pex1*^+/+^ (WT), *pex1*^+/-^ (Het), and *pex1*^-/-^ (Homo) zebrafish. Fatty acid levels were normalized to C20:0 and expressed relative to the average WT level for each fatty acid. Log2 ratios are presented as mean ± SD (n = 3 per genotype). Statistical significance was evaluated by two-way ANOVA (**p* ≤ 0.05, **p ≤ 0.002, ***p ≤ 0.0002, ****p ≤ 0.0001).

**Supplementary Figure 3**. VLCFA levels in livers of 7-month-old *pex1*^+/+^ (WT), *pex1*^+/-^ (Het), and *pex1*^-/-^ (Homo) zebrafish. Fatty acid levels were normalized to C20:0 and expressed relative to the average WT level for each fatty acid. Log2 ratios are presented as mean ± SD (n = 3 per genotype). Statistical analysis was performed using two-way ANOVA (*p ≤ 0.05, **p ≤ 0.002, ***p ≤ 0.0002, ****p ≤ 0.0001).

Supplementary Figure 4. Fatty acid content of zebrafish dry food and live feed. (A) Comparison of DHA levels across different zebrafish food sources. (B) Fatty acid levels (μg/mg food) in SDS100, SDS400, artemia, rotifers, and algae. Results were obtained from two independent analyses. Means are shown in panels A and B, and replicate measurements (dots) are also shown in panel A.

**Supplementary Figure 5.** Targeted lipidomics analysis in 13 dpf *pex1*^+/+^ (WT) and *pex1*^-/-^ (Homo) zebrafish larvae. (A) Stacked bar plot showing levels of triacylglycerol fatty acids (FAs) grouped by carbon chain length or degree of saturation. (B) Stacked bar plot showing the relative abundance of individual FA species within the triacylglycerol fraction. (C) Comparison of the levels of additional lipid classes between WT and Homo larvae. For each class, values represent the sum of mean FA levels across five biological replicates, each consisting of a pool of 15 larvae at 13 dpf. FA levels were normalized to DNA content. Error bars indicate SDs. Statistical significance was determined using unpaired Welch’s *t*-tests. *p<0.033; ***p<0.0002; ****p< 0,0001; ns, not significant; DG, diacylglycerides; CE, cholesterol esters; CER, ceramides; Lac-Cer, lactosyl-ceramides; LPE, lysophosphatidylethanolamines; PI, phosphatidylinositols; PG, phosphatidylglycerols; PS, phosphatidylserines; LPC, lysophosphatidylcholines; PE, phosphatidylethanolamines.

**Supplementary Figure 6.** Visualization of high-ranked pathways obtained in transcriptomics analysis of 13 dpf *pex1*^+/+^ and *pex1*^-/-^ zebrafish larvae. Map for Apoptosis_Endoplasmatic reticulum stress pathway. Significant up-regulation of genes is indicated as up-pointing bars colored in red. The length of the colored bar is proportional to the -fold change of gene expression in the meta-analysis.

­­­­

**Supplementary Figure 7.** Visualization of high-ranked pathways obtained in transcriptomics analysis of 13 dpf zebrafish larvae. Map for Neurophysiological process_visual perception. DEGs are indicated in red (upregulated in *pex1*^-/-^ larvae) and blue circles (downregulated in *pex1*^-/-^ larvae). Green, red, and gray arrows indicate positive, negative, and unspecified effects, respectively.

**Supplementary Figure 8.** Characterization of locomotor activity, body size, and eye area in *pex1*^+/+^ (WT) and *pex1*^-/-^ (Homo) zebrafish larvae. (A) Locomotor activity of larvae at the indicated developmental stage, measured as mean velocity (1-min intervals) under constant light conditions. (B, C) Body length and eye area measurements at 13 dpf, showing no significant differences between genotypes. (D) Behavioral activity profiles of 7 and 15 dpf larvae exposed to alternating light/dark cycles (10-min intervals), measured in 1-min bins. (E) Startle response profile (1-sec resolution) at 13 dpf under acoustic stimulation, with stimuli applied every 30 seconds. In panels (A), (D), and (E), each dot represents the mean velocity of 24–36 larvae recorded across two independent plates; error bars indicate SEMs. In panels (B) and (C), each dot represents an individual larva and the horizontal bar shows the mean.

**Supplementary Data 1.** Excel file containing multiple sheets with the following information: qPCR primer sequences; list of antibodies used for immunohistochemistry experiments; raw data from transcriptomic analyses; raw data from VLCFA measurements in adult and larval zebrafish, as well as in zebrafish food; raw data from the targeted lipidomics analyzes performed in larval extracts.
